# The myokine FGF21 associates with enhanced survival in ALS and mitigates stress-induced cytotoxicity

**DOI:** 10.18632/aging.206298

**Published:** 2025-08-09

**Authors:** Abhishek Guha, Ying Si, Reed Smith, Brijesh K. Singh, Benisa Zogu, Angad Yadav, Katherine A. Smith, Mohamed Kazamel, Nan Jiang, Ritchie Ho, Anna Thalacker-Mercer, Shaida A. Andrabi, Joao D. Tavares Da Silva Pereira, Juliana S. Salgado, Manasi Agrawal, Emina Horvat Velic, Peter H. King

**Affiliations:** 1 Department of Neurology, University of Alabama at Birmingham, Birmingham, AL 35294, USA; 2 Birmingham Veterans Affairs Medical Center, Birmingham, AL 35294, USA; 3 Killion Center for Neurodegeneration and Experimental Therapeutics, University of Alabama at Birmingham, Birmingham, AL 35294, USA; 4 Board of Governors Regenerative Medicine Institute, Cedars-Sinai Medical Center, Los Angeles, CA 90048, USA; 5 Center for Neural Science and Medicine, Cedars-Sinai Medical Center, Los Angeles, CA 90048, USA; 6 Department of Biomedical Sciences, Cedars-Sinai Medical Center, Los Angeles, CA 90048, USA; 7 Department of Cell, Developmental, and Integrative Biology, University of Alabama at Birmingham, Birmingham, AL 35294, USA; 8 Department of Neurology, Cedars-Sinai Medical Center, Los Angeles, CA 90048, USA; 9 Department of Neuroscience, Yale University School of Medicine, New Haven, CT 06510, USA

**Keywords:** fibroblast growth factor 21, β-Klotho, ALS biomarker, human skeletal muscle, motor neurons

## Abstract

Amyotrophic lateral sclerosis (ALS) is an age-related and fatal neurodegenerative disease characterized by progressive muscle weakness. There is marked heterogeneity in clinical presentation, progression, and pathophysiology with only modest treatments to slow disease progression. Molecular markers that provide insight into this heterogeneity are crucial for clinical management and identification of new therapeutic targets. In a prior muscle miRNA sequencing investigation, we identified altered FGF pathways in ALS muscle, leading us to investigate FGF21. We analyzed human ALS muscle biopsy samples and found a large increase in FGF21 expression with localization to atrophic myofibers and surrounding endomysium. A concomitant increase in FGF21 was detected in ALS spinal cords which correlated with muscle levels. FGF21 was increased in the SOD1^G93A^ mouse beginning in presymptomatic stages. In parallel, there was dysregulation of the co-receptor, β-Klotho, with higher levels detected in ALS muscle biopsies and lower levels in post-mortem muscle compared to controls. Plasma FGF21 levels were increased in ALS patients and high levels correlated with slower disease progression, prolonged survival, and increased body mass index. *In cellulo*, FGF21 was induced in differentiating muscle cells and ectopic treatment with FGF21 enhanced muscle differentiation. Ectopic FGF21 mitigated oxidative stress-induced loss of viability in iPSC-derived ALS motor neurons and muscle cells expressing SOD1^G93A^. In summary, FGF21 is a novel biomarker in ALS which exerts trophic effects in the neuromuscular system.

## INTRODUCTION

Amyotrophic lateral sclerosis (ALS) is a multisystemic and age-related neurodegenerative disease characterized by progressive weakness from degeneration of the motor axis including upper and lower motor neurons. This weakness ultimately leads to respiratory failure and death approximately 3 to 5 years after onset [1, 2]. The neuromuscular component of the motor axis plays a pivotal role in progression of ALS, with some investigators postulating a direct role in disease initiation [3, 4]. Indeed, insights from animal studies reveal that initial pathological changes in ALS manifest peripherally, characterized by the breakdown of the neuromuscular junction (NMJ) and the presence of mitochondrial abnormalities within skeletal muscle [5, 6]. We and others have characterized a dysregulated transcriptome in skeletal muscle of ALS patients (see recent review [4]). Our group has identified aberrant expression of proteins and non-coding RNAs reflecting different signaling pathways such as TGFβ/Smad, Wnt, vitamin D and FGF23 [7–13]. Interestingly, many of these molecular patterns overlap in the SOD1^G93A^ mouse, often starting in the early pre-clinical stages and changing with disease progression [4]. Some of the biomarkers identified contribute to an intricate regulatory network, facilitating molecular crosstalk between motor neurons and myofibers to support the maintenance and regenerative capacity of the neuromuscular unit. On the other hand, other factors may be detrimental to the motor axis [3, 4, 14]. Hence, characterizing these biomarkers and exploring the molecular pathways they reflect can lead to improved patient management, including clinical trials, while also identifying novel targets for therapeutic development [15].

In a prior miRNA sequencing analysis with ALS muscle tissue, we found patterns predicted to alter FGF21 signaling [12]. Based on this background, we hypothesized that FGF21 may be dysregulated in ALS skeletal muscle early on and serve as a biomarker. FGF21 is a hormone primarily produced in the liver under physiological states and plays a major role in regulating glucose and energy metabolism [16]. In stressed or pathophysiological conditions, such as mitochondrial stress/dysfunction or endoplasmic (ER) stress, FGF21 can be induced in many tissues, including skeletal and cardiac muscle, where it can mitigate metabolic dysfunction and restore and/or maintain mitochondrial homeostasis through autocrine and paracrine pathways [17–20]. It has been proposed as a biomarker of healthy aging and has been shown in animal models to extend lifespan [21–23].

Here, we utilized an extensive human repository of ALS tissue to investigate FGF21 and found a significant increase of FGF21 expression in ALS muscle, predominantly in atrophied myofibers and surrounding endomysial connective tissue. We found a concomitant increase in the mRNA of the co-receptor, β-Klotho (*KLB*), suggesting that FGF21 signaling is increased in the neuromuscular unit. FGF21 was increased in plasma samples of ALS patients, and high levels associated with slower disease progression and longer survival. Using *in cellulo* models, we found that ectopic FGF21 exerts a trophic effect in oxidatively stressed ALS motor neurons, normal human muscle cells, and C2C12 muscle cells expressing SOD1^G93A^. In myoblasts, FGF21 was induced upon differentiation and promoted myogenesis.

## MATERIALS AND METHODS

### Human clinical samples collection

Muscle samples and post-mortem tissues were obtained under UAB Institutional Review Board (IRB)-approved protocols as described previously [7, 8, 24]. Some of the post-mortem spinal cord samples (normal and ALS) were provided by the Department of Veterans Affairs Biorepository (VA Merit review BX002466).

Blood samples were collected from non-fasting ALS patients and normal controls enrolled in a prior biomarker study approved by the UAB IRB [24]. Samples were drawn in EDTA plasma collection tubes, centrifuged at 1600 RCF for 10 min at 4° C, and stored at -80° C. For correlation studies with disease progression and survival, enrolled patients had a sample at study entry and 3 months later.

### Animal sample collection

All animal procedures were approved by the UAB Institutional Animal Care and Use Committee and were carried out in accordance with relevant guidelines and regulations of the National Research Council Guide for the Care and Use of Laboratory Animals and in compliance with the ARRIVE guidelines. B6.Cg-Tg (SOD1_G93A) 1 Gur/J male mice (Jackson Laboratory, ME, USA) were generated as previously detailed [8]. After euthanasia, gastrocnemius muscle and spinal cord tissue samples were collected from SOD1^G93A^ and WT-SOD1 littermate controls at post-natal day 20, 40, 60, 125 and 150. These time points cover the full range of disease stages in the ALS mouse as previously described [11].

### Cell culture, treatment, and transfection

C2C12 myoblasts (ATCC, VI, USA) and NSC-34 cells were grown in 0.22 μm filter (Corning, NY, USA) sterilized Dulbecco’s modified Eagle’s High Glucose (Corning) pH 7.4, supplemented with 10% FBS (Thermo Fisher Scientific, MA, USA), 1% PEN-STREP (Thermo Fisher Scientific) and 1% L-Glutamine at 37° C in a humidified 5% CO_2_ incubator. Human Skeletal Muscle Myoblasts (HSMM; Lonza, Switzerland) primary cells were cultured in skeletal muscle cell growth medium (Ready-to-use), comprising basal medium (Sigma Aldrich, MO, USA) and SupplementMix (PromoCell, Germany, C-39365), following the manufacturer's instructions. C2C12 and NSC-34 cells were treated with methionine-cystine (MetCys)-deprived media (Thermo Fisher Scientific) or 100 ng/ml mouse recombinant FGF21 (GenScript, NJ, USA) or exposed to 100 μM H_2_O_2_ (Sigma Aldrich) for 24 h. Additionally, some of these cells were transfected with pCDNA3.1 FGF21-Flag (GenScript), Flag-SOD1^G93A^ and Flag-SOD1WT plasmid constructs [25] using Lipofectamine 2000 (Thermo Fisher Scientific) in Opti-MEM Reduced Serum Media (Thermo Fisher Scientific). HSMM cells were treated with 100 ng/ml human recombinant FGF21 (GenScript) or exposed to 25 μM H_2_O_2_ (Sigma Aldrich) for 24 h. To induce C2C12 myoblast differentiation, growth media (GM) was switched to differentiation media (DM) containing 2% horse serum. For HSMM myogenic differentiation, cells were maintained in skeletal muscle cell DM (Ready-to-use), containing basal medium (Sigma Aldrich) and SupplementMix (PromoCell, C-39366).

### iPSC-derived motor neurons

iPSCs were derived from ALS subjects and controls and are summarized in Supplementary Table 1. Differentiation of motor neurons followed methods previously published by our group [11] except for the ALS lines, NN0004306 and NN0004307, and the control lines, FA0000011 and NN0003920. For these four lines, a different protocol was used and detailed elsewhere [26, 27]. iPSC motor neurons were treated with different doses of H_2_O_2_ (0-100 μM) and human recombinant FGF21 (0-50 ng/ml) for 24 h.

### Cell proliferation and cell viability assay

C2C12 cells were transfected with a FLAG-tagged-FGF21 expressing plasmid in a dose-dependent manner, and after 48 h post-transfection, cells were (1 x 10^4^) seeded in 96-well plate. Cell proliferation was assessed at intervals of 24, 48, and 72 h using the MTS-based cell growth determination kit (Promega, WI, USA). C2C12, HSMM, NSC-34 cells, and iPSC motor neurons were subjected to oxidative stress via MetCys-deprived media and/or H_2_O_2_ treatment, and rescue experiments were performed using mouse or human recombinant FGF21, as described before. Cell viability was determined with the ViaLight Plus BioAssay kit (Lonza) [28], MTS-based cell viability kit (Promega) and cell counting kit-8 (Dojindo Laboratories, Japan) following the manufacturers' instructions. Apoptosis was detected by caspase activity using Caspase-Glo 3/7 assay kit (Promega) following the manufacturer's protocol.

### RNA analysis

RNA was extracted from muscle and spinal cord tissue samples using TRIzol (Thermo Fisher Scientific) and RNASpin Mini kit (Cytiva, MA, USA) per the manufacturer’s instructions. RNA from iPSC motor neurons was extracted using the RNeasy kit (Qiagen, NJ, USA) following the manufacturer's instructions. For other cell culture samples, cells were lysed using the lysis buffer provided in the RNASpin Mini kit. cDNAs were synthesized using the High-Capacity cDNA Reverse Transcription Kit (Thermo Fisher Scientific). mRNA expression of *FGF21* and *KLB* was quantified utilizing Taqman primers (Thermo Fisher SCientific) with the QuantStudio 5 Real-Time PCR system (Thermo Fisher SCientific). *RPS9* and *TBP1* were used as internal controls. For iPSC-derived motor neurons, a fold-change in RNA for ALS motor neurons was generated by comparing to control lines within the same differentiation protocol.

### Immunoblotting and immunochemistry

Immunoblotting was performed based on methods described previously [8]. The following antibodies were used: anti-FGF21 (Abcam, UK), 1:1000; anti-MHC (Developmental Studies Hybridoma Bank), 1:200; anti-FLAG-M2 (Sigma Aldrich), 1:1000; anti-Vinculin (Abcam), 1:3000.

For immunocytochemistry, C2C12 (1 x 10^5^) and HSMM (6 x 10^4^) cells were seeded in Nunc Chamber Slide System (Thermo Fisher Scientific) and then treated with DM 24 h later. Cells were fixed at different time intervals and blocked using Intercept (PBS) Blocking Buffer (Li-COR) for 30 min. Subsequently, cells were stained using an anti-MHC antibody (Developmental Studies Hybridoma Bank), 1:50, overnight at 4° C, followed by an anti-Mouse Alexa Fluor 488 secondary antibody (Thermo Fisher Scientific), 1:400. Nuclei were stained with DAPI (Thermo Fisher Scientific) at 1:2000 for 5 min at RT. Images were captured using a Nikon C2 confocal microscope. The fusion index was calculated as the number of nuclei inside MHC-positive myotubes divided by the total number of nuclei present in a field of view from three random fields per biological replicate (total of 3 replicates) [29].

For immunohistochemistry, muscle samples were fixed in 4% PFA at 4° C overnight and embedded in paraffin. Deparaffinized 10 μm sections were boiled in 10 mM citrate buffer (pH 6.0) for 30 min, and cooled to RT. Slides were incubated in 3% H_2_O_2_ for 10 min and then incubated with FGF21 antibody (Abcam), 1:100, overnight at 4° C. After washing in PBS, slides were incubated with a donkey anti-rabbit Cy3 secondary antibody (Jackson Immunoresearch, PA, USA) for 90 min at RT. Sections were incubated in Wheat Germ Agglutinin (WGA), Oregon Green 488 Conjugate (Thermo Fisher Scientific), followed by DAPI (Thermo Fisher Scientific) at 1:2000 for 5 min. Confocal imaging was done to assess atrophic and non-atrophic muscle fibers. Mean FGF21 fluorescence intensity (MFI) was assessed on muscle tissue samples as described previously [7, 24]. The minimal Feret’s diameter of selected myofibers determined myofiber size, with fibers less than 25 μm in diameter considered atrophic. A total of 46 atrophic fibers were compared to an equal number of non-atrophic fibers within the same images. Image analysis was done by the investigators blinded to the identity of the samples.

### ELISA

FGF21 was quantitated in muscle, spinal cord and plasma samples using U-PLEX Human FGF21 ELISA Assay (K1515WK-1, MSD) according to the manufacturer’s instruction. For survival and rate of disease progression, the FGF21 level at study entry and the 3-month time interval were averaged. To evaluate the levels of secreted FGF21 protein, conditioned media (CM) were collected from C2C12 and NSC-34 cells following exposure to different treatments or conditions. FGF21 levels were quantitated using U-PLEX Mouse FGF21 Assay (K1525WK-1, MSD). A limitation of the ELISA is that it cannot distinguish between intact and cleaved FGF21 in human plasma.

### Statistics

Statistical analyses for both human and mouse data were conducted using GraphPad Prism 10.2 (San Diego, CA, USA). *FGF21* mRNA expression in human muscle biopsy samples was analyzed with a two-tailed t-test with a Welch’s correction. For *KLB* mRNA, a two-tailed Mann Whitney test was used for comparison. A two-tailed Mann Whitney test was used for post-mortem tissues, FGF21 quantitative immunostaining, and iPSC-derived motor neurons. A two-tailed t-test was used for mouse muscle and spinal cord tissue and cell culture analyses, plasma FGF21, BMI, viability, and Caspase-3/7 activity. One-way ANOVA with Tukey’s multiple comparisons test was used for comparisons of plasma FGF21 levels between different disease progression subsets, cell stress-induced changes in viability, and C2C12 proliferation assays. A Spearman correlation test was used for spinal cord versus muscle FGF21 protein levels, muscle *FGF21* versus *HDAC4* mRNA levels, and ∆ALSFRS versus plasma FGF21 levels. A Log-rank (Mantel-Cox) test was used to compare Kaplan Meier survival curves in ALS patients.

## RESULTS

### FGF21 is increased in human ALS muscle and spinal cord tissue

**Figure 1:**
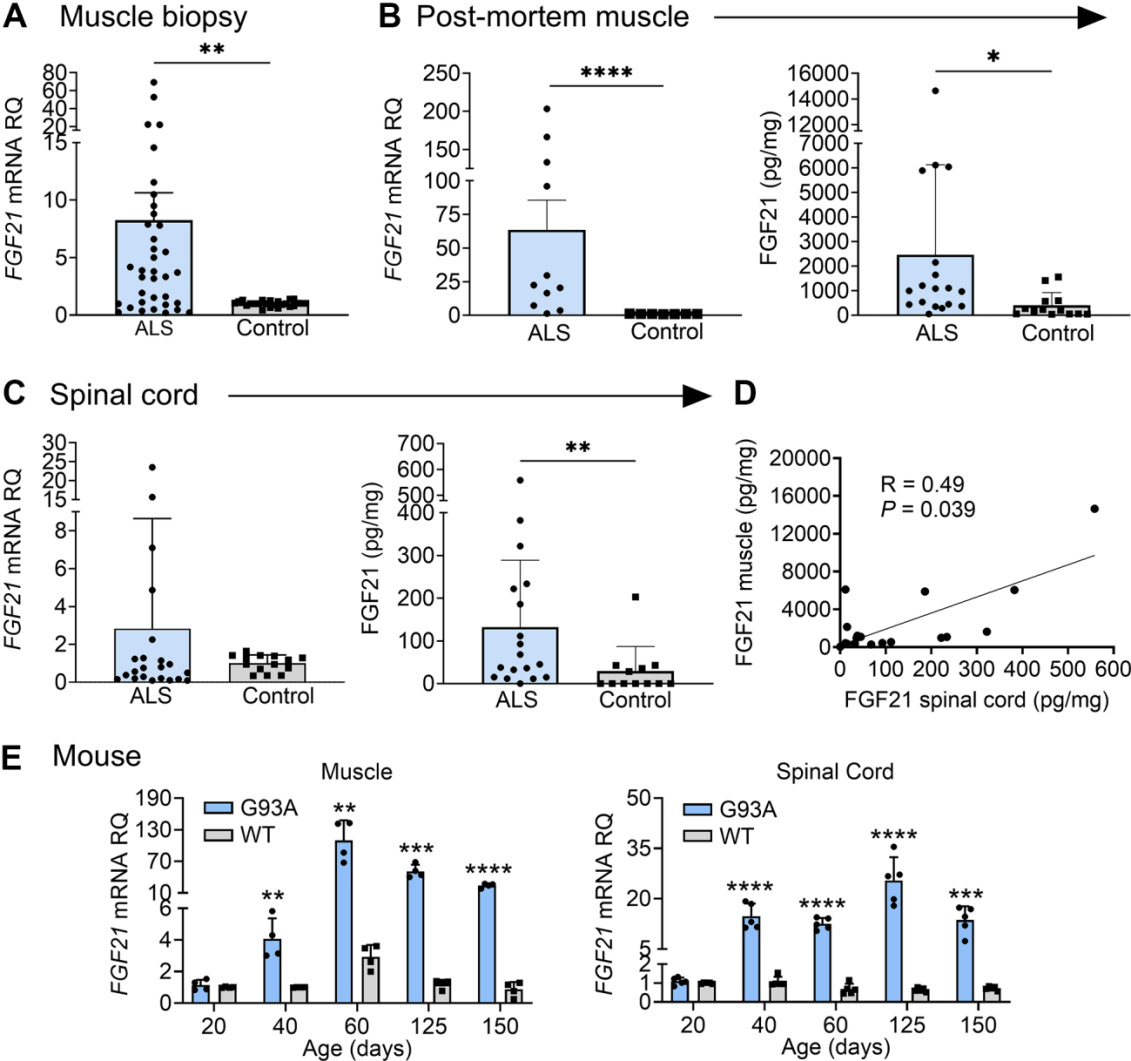
**FGF21 levels are elevated in the muscle and spinal cord tissues of ALS patients and the SOD1^G93A^ mouse.** (**A**) *FGF21* mRNA expression was analysed in normal (*n* = 24) and ALS (*n* = 36) muscle biopsy samples via RT-qPCR. **P = 0.004, unpaired two-tailed t-test with Welch’s correction. (**B**) *FGF21* mRNA levels were quantified in normal (*n* = 7) and ALS (*n* = 11) post-mortem muscle samples (left panel) and FGF21 protein levels (*n* = 13 for normal samples; *n* = 18 for ALS samples; right panel). **P = 0.003, ****P < 0.0001, two-tailed Mann Whitney test. (**C**) *FGF21* mRNA levels were quantified in normal (*n* = 14) and ALS (*n* = 22) post-mortem spinal cord samples (left panel) and FGF21 protein levels (*n* = 12 for normal samples; *n* = 18 for ALS samples; right panel). **P = 0.00, two-tailed Mann Whitney test. (**D**) Comparison of spinal cord and muscle FGF21 protein levels for 18 ALS patients. A spearman correlation test was used for analysis. (**E**) *FGF21* mRNA levels in the gastrocnemius muscle (left panel) and spinal cord (right panel) were quantified across different age groups (20 – 150 days; *n* = 4-5 per group) from SOD1^G93A^ mice and littermate controls. **P < 0.01, ***P < 0.001, ****P < 0.0001, unpaired two-tailed t-test comparing WT to SOD1^G93A^. For all graphs, error bars represent SD.

We assessed *FGF21* mRNA expression in a large cohort of muscle biopsy samples of ALS patients from our clinic (Supplementary Table 2 and Figure 1). Demographics for this cohort, including age (mean 57 ± 13 y) and slight male to female predominance (1.4:1), were in line with prior epidemiological studies [1, 30]. The cohort of normal biopsies showed similar age with a slight female to male predominance (1.2:1). By qPCR, we found an ~8-fold increase in *FGF21* mRNA in ALS samples (*P =* 0.004) compared to normal control biopsy samples (Figure 1A). There was variability in mRNA levels with several at greater than 50-fold higher than controls. We next tested post-mortem ALS muscle samples which represent more advanced (end-stage) disease as reflected by the longer disease duration (51 months versus 15 months in the biopsy group). *FGF21* mRNA levels were much higher at 63-fold greater than normal control samples, indicating that *FGF21* expression in muscle increases with disease progression (Figure 1B). We compared *FGF21* mRNA levels with *HDAC4*, another known marker of muscle denervation in ALS [31], and found suggestive evidence of a correlation (Spearman rank correlation *R* = 0.683, *P* = 0.050, Supplementary Figure 1). We next assessed FGF21 protein expression by ELISA with post-mortem samples and found a ~7-fold increase in ALS samples (2463 versus 335 pg/mg of muscle tissue; *P =* 0.030). As with mRNA levels, there was variability among ALS samples (versus controls) with one ALS patient showing nearly 15,000 pg/mg. We next measured *FGF21* mRNA levels in spinal cord tissue but observed no significant difference compared to control tissue as a whole; however, there were 4 outliers with markedly increased levels of up to 25-fold (Figure 1C). On the other hand, FGF21 protein was increased in ALS spinal cord (132 versus 29 pg/mg of spinal cord tissue; *P =* 0.005), but levels in spinal cord were nearly 19-fold less than ALS muscle. There was variability among the ALS patients as observed with muscle. Post-mortem spinal cord and muscle samples were matched for 18 of the ALS patients tested and they showed a positive correlation for FGF21 levels (Spearman rank correlation *R* = 0.49, *P =* 0.039) (Figure 1D). We next sought to determine whether changes in *FGF21* occurred in the SOD1^G93A^ mouse model of ALS. This model can provide insight into temporal patterns of biomarkers identified in human ALS muscle including pre-clinical (prodromal) phases of the disease [4]. We detected a 4-fold increase in *FGF21* mRNA compared to age-matched wild-type (WT) controls at 40 d post-natal (Figure 1E). Although NMJ innervation is reduced by 40% by this age, this stage is typically considered pre-symptomatic where standard testing such as rotarod and grip strength are unaffected and only subtle signs of muscle weakness can be detected by more in-depth testing [32]. In later stages, muscle FGF21 rose from ~37-fold at 60 d (pre-symptomatic) to 43-fold at 125 d (symptomatic stage). At end-stage (150 d), there was a 28-fold fold-change. FGF21 mRNA increased in spinal cord tissue in parallel with muscle FGF21. Taken together, FGF21 mRNA and protein levels were significant but variably increased in human ALS muscle and spinal cord tissues, with a disproportionate increase in muscle. Furthermore, the strong association between muscle and spinal cord FGF21 for each patient and in the SOD1^G93A^ mouse model suggested an underlying connectivity.

### FGF21 localizes to atrophic muscle fibers

**Figure 2:**
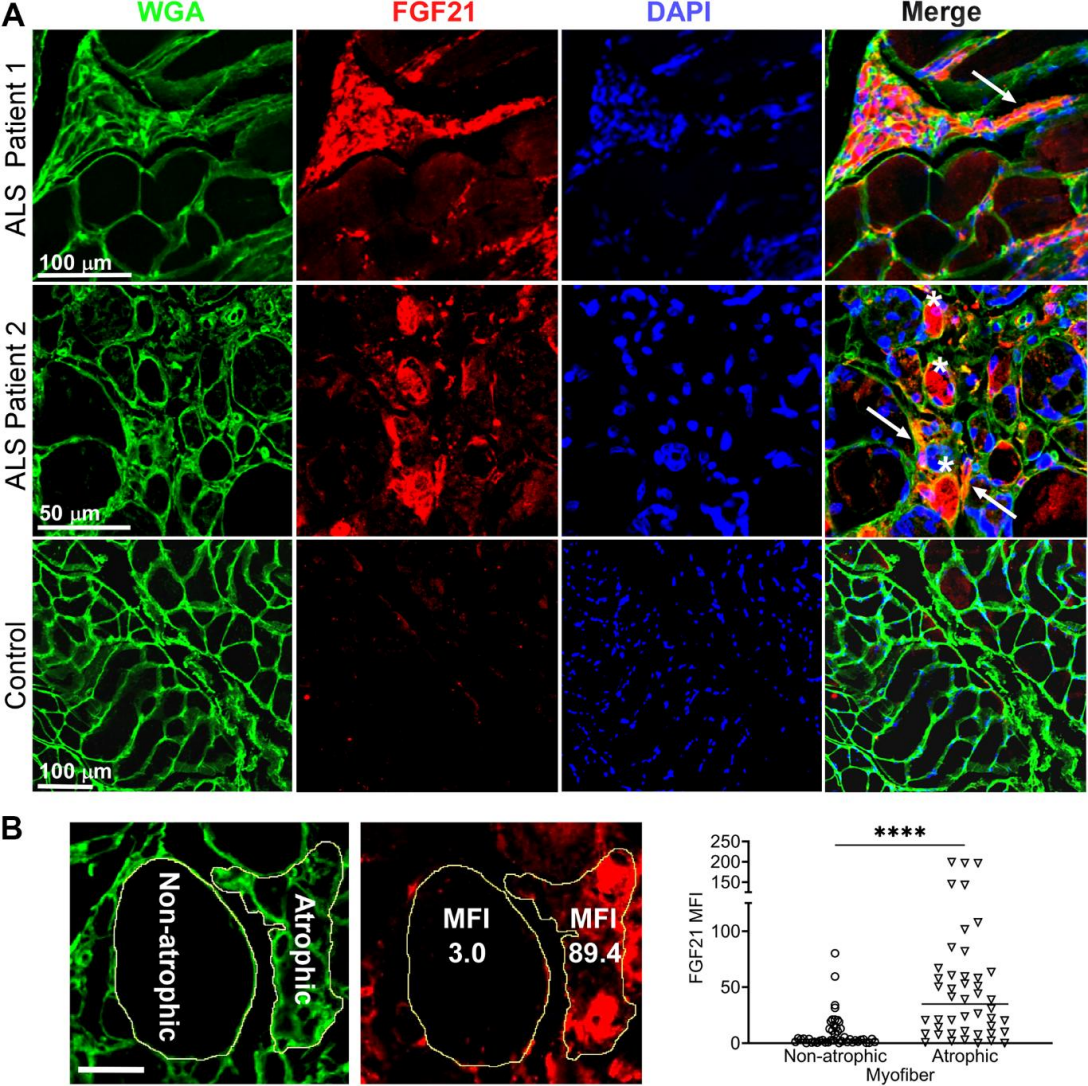
**FGF21 localizes to atrophic myofibers in ALS muscle.** (**A**) Tissue sections from two ALS patients and one normal control were immunostained with an anti-FGF21 antibody and counterstained with DAPI and wheat germ agglutin (WGA). Intense immunoreactivity is observed in atrophic myofibers (asterisks) and in the endomysial space (arrows) in the ALS muscle sections. Scale bars, 100 μm in low power views and 50 μm in the enlarged views. (**B**) Mean fluorescence Intensity (MFI) analysis of FGF21 immunoreactivity was performed in 5 ALS patient biopsy samples and 5 normal controls. Atrophic (< 25 μm minimal Feret’s diameter) and non-atrophic myofibers were selected in the same section as shown in the micrograph (yellow outline). FGF21 MFI (per μm^2^) was quantitated for 46 atrophic and non-atrophic myofibers and summarized in the graph (horizontal line represents the mean). ****P < 0.0001; two-tailed Mann Whitney test. Scale bar: 50 μm.

Next, we determined localization of FGF21 in ALS muscle by immunohistochemistry. We observed intense intra-myofiber staining in areas of grouped atrophy (characterized by clusters of small angular fibers) which is a hallmark of denervation (Figure 2A and Supplementary Figure 7) [33]. FGF21 staining also localized to the endomysial connective tissue. On the other hand, non-atrophic fibers within the same section showed little or no staining. Five normal control muscle samples also showed no FGF21 staining (example shown in Figure 2A and Supplementary Figure 7). To confirm an association between FGF21 immunoreactivity and atrophic myofibers, we measured mean fluorescence intensity (MFI) in atrophic and non-atrophic fibers in muscle sections from five ALS patients (Figure 2B and Supplementary Figure 7). Regions of interest (ROI) for atrophic and non-atrophic myofibers were selected within the same muscle section, often in proximity, to minimize the effects of variable tissue staining (see example in Figure 2B and Supplementary Figure 7). We observed a 9-fold increase in FGF21 MFI in ROIs associated with atrophic versus non-atrophic myofibers (*P* < 0.0001). Taken together, these data indicate a marked increase in FGF21 expression in ALS muscle tissues, predominantly in atrophic myofibers and surrounding endomysial connective tissue.

### Plasma FGF21 levels are elevated in ALS patients and correlate with enhanced survival

**Figure 3:**
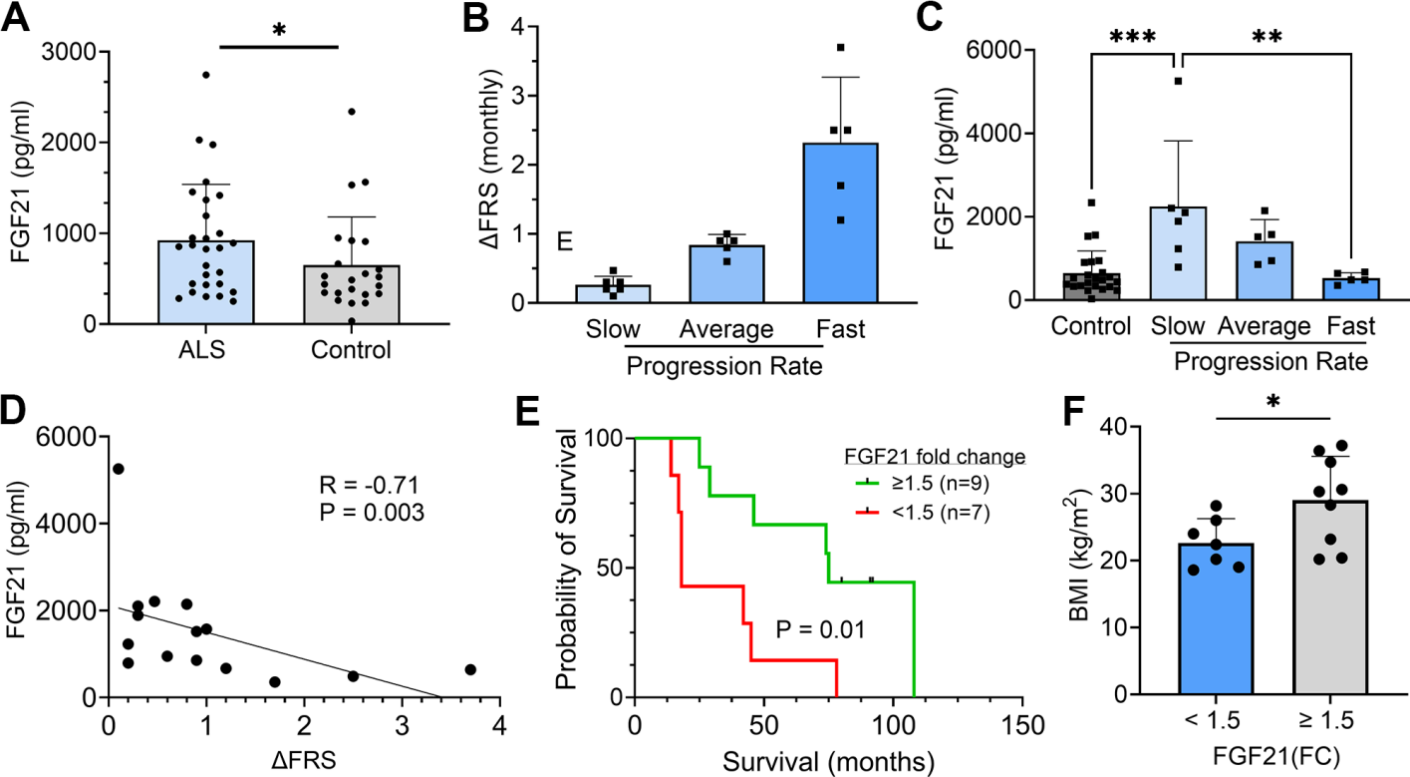
**Plasma FGF21 is increased in ALS patients and high levels associated with slower disease progression and prolonged survival.** (**A**) Plasma samples from age-matched normal controls (*n* = 23) and ALS patients (*n* = 28) and assayed by ELISA for FGF21. *P = 0.043, unpaired two-tailed t-test. (**B**) 16 ALS patients from a prior biomarker study were divided into slow (*n* = 6), average (*n* = 5), and fast (*n* = 5) progressing groups based on the average in the study monthly decline in ALSFRS-R scores. (**C**) Plasma FGF21 levels were measured at baseline and 3 months and averaged. The normal control values were added for comparison. **P = 0.003, ***P = 0.0003; one-way ANOVA followed by Tukey post hoc test. (**D**) Correlation between plasma FGF21 levels with monthly change in the ALSFRS-R scores (∆FRS) for each subject. Spearman correlation test. (**E**) Kaplan–Meier survival curves for study patients whose FGF21 plasma levels were < 1.5-fold-change (FC) over the normal control group versus study patients with ≥ 1.5-FC in FGF21 levels. *P = 0.015; Log-rank (Mantel-Cox) test. (**F**) Comparison of body mass index (BMI) between the < 1.5 FC and ≥ 1.5-FC groups. *P = 0.037; unpaired two-tailed t-test. For all graphs, error bars represent SD.

Since FGF21 is a secreted factor and we observed extra-myofiber immunostaining in ALS muscle tissue, we queried whether FGF21 could be detected in plasma of ALS patients. To assess this possibility, we assayed plasma samples collected from a cohort of ALS patients in our clinics who participated in a prior biomarker study (Supplementary Table 3) [9]. A set of age-matched normal controls was used for comparison. Overall, we found a significant increase in FGF21 levels in the ALS cohort (923 v. 649 pg/ml; *P* = 0.03), but with variability (Figure 3A). Interestingly, the FGF21 levels were more than 2-fold higher in ALS muscle versus plasma FGF21 (albeit end-stage ALS muscle; Figure 1B). None of the patients or controls had type II diabetes or known liver disease, two conditions associated with higher circulating levels of FGF21 [34]. We next examined a subset of 16 ALS patients who were followed prospectively with serial examinations and plasma sample collection. Based on calculated monthly changes in ALSFRS-R scores (∆FRS), there were 6 slow, 5 average and 5 fast progressors using previously established criteria (Figure 3B) [35]. Using the average of baseline and 3-month FGF21 plasma levels, we found that the slow progressors had more than a 4-fold increase in FGF21 compared to fast progressors (2247 v. 529 pg/ml; P = 0.003) and a ~3.5-fold increase compared to the control group (P = 0.0003; Figure 3C). The group of average progressors was intermediate between the slow and fast groups (1410 pg/ml) but did not reach statistical significance. Independent of clinical classification, there was a strong negative correlation between the ∆FRS for each subject and their respective plasma FGF21 level (*R* = -0.710, *P* = 0.003; Figure 3D). We then assessed survival of these patients based on FGF21 levels, comparing those with <1.5 fold-change (FC) over controls versus ≥ 1.5 FC (Figure 3E). The median survival for the group with low FGF21 levels was 18 months versus 75 months for the group with high FGF21 levels (*P* = 0.01). Three patients in the high FGF21 group are still alive at 82, 93, and 94 months after disease onset. We next looked at body mass index (BMI) and found that the <1.5 FC group had a significantly lower BMI than the >1.5 FC group (22.6 ± 3.6 v. 29.0 ± 6.6 kg/m^2^, *P* = 0.04; Figure 3F). Age and duration of disease were not significantly different between the two groups (Supplementary Table 4). Interestingly, in the lower FGF21 group, 4 out of 7 patients had bulbar onset of ALS versus the high FGF21 group which all had spinal onset. Since liver is a major source of FGF21 [36], we assessed expression in the SOD1^G93A^ mouse at a time point of peak FGF21 expression in skeletal muscle (Supplementary Figure 2). Similar to results in Figure 1, we observed a ~150-fold increase in FGF21 mRNA in the SOD1^G93A^ mouse compared to WT control muscle (set at 1). In liver, the increase was significantly less at 75-fold in the SOD1^G93A^ mouse (*P* < 0.0001) although the mRNA level in WT liver was ~35-fold greater than WT muscle. Liver tissue was not available in our post-mortem repository to confirm whether this discrepancy applies more broadly to ALS patients. Taken together, ALS patients had an overall increase in plasma FGF21 and high levels correlated with slower disease progression and longer survival. While FGF21 mRNA is increased in muscle and liver tissues in the SOD1^G93A^ mouse, the disproportionately higher increase in muscle suggests that this tissue is a major contributor to circulating FGF21.

### FGF21 co-receptor, β-Klotho, is altered in ALS muscle and spinal cord tissue

**Figure 4:**
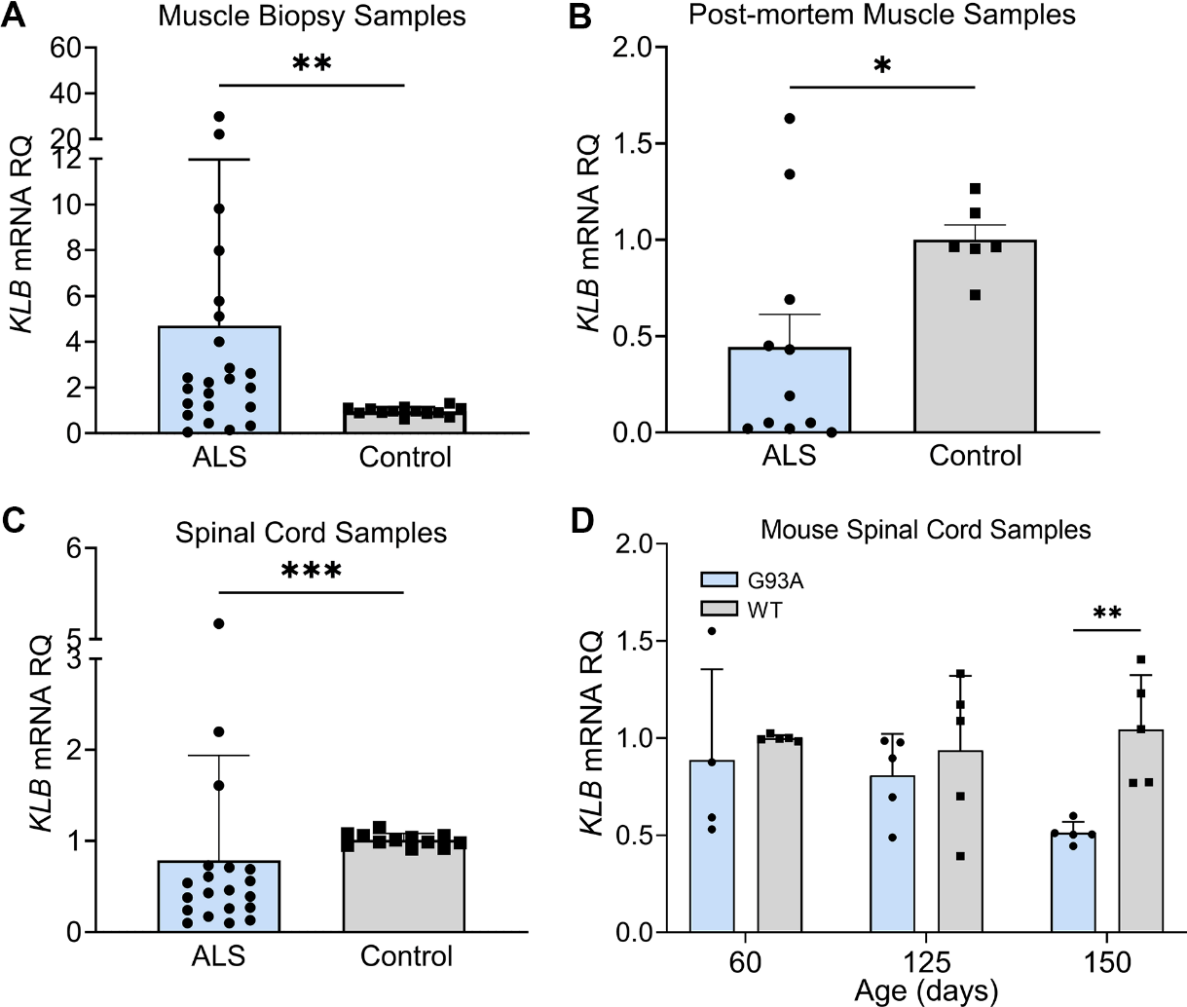
**FGF21 coreceptor, *β-Klotho* (*KLB*), is dysregulated in ALS muscle and spinal cord.** (**A**) *KLB* mRNA levels were measured in muscle biopsy samples from normal (*n* = 13) and ALS patients (*n* = 23). **P = 0.005, two-tailed Mann Whitney test. (**B**) *KLB* mRNA levels were measured in post-mortem muscle samples from normal controls (*n* = 6) and ALS (*n* = 11) patients. *P = 0.034, two-tailed Mann Whitney test. (**C**) *KLB* mRNA levels were measured in spinal cord samples from normal controls (*n* = 12) and ALS patients (*n* = 20). ***P = 0.0006, two-tailed Mann Whitney test. (**D**) *KLB* mRNA levels were measured in spinal cord samples from SOD1^G93A^ mice (*n* = 5) and WT controls (*n* = 5) at different ages. **P = 0.003; unpaired two-tailed t-test comparing WT to SOD1^G93A^.

Given the critical role of β-Klotho (KLB) co-receptor in FGF21 signaling [36], we assessed *KLB* mRNA expression in muscle biopsy samples and found a ~ 4-fold increase in *KLB* levels in the ALS group versus controls (Figure 4A; *P =* 0.005). On the other hand, post-mortem ALS muscle samples had a 50% reduction in *KLB* mRNA levels (*P =* 0.03) compared to normal controls (Figure 4B). Likewise, ALS spinal cord tissue showed a significant reduction in *KLB* levels (*P =* 0.0006) compared to controls (Figure 4C). Some variability was observed with three samples having increased mRNA. Spinal cord tissue from end-stage SOD1^G93A^ mice paralleled these findings and showed a two-fold reduction of *KLB* mRNA compared to littermate controls (*P =* 0.008; Figure 4D). Collectively, these findings suggest a dysregulation of *KLB* expression in ALS muscle and spinal cord tissues that changed with disease stage and may relate to a progressive loss of motor neurons.

### FGF21-KLB axis is dysregulated in ALS motor neurons

**Figure 5:**
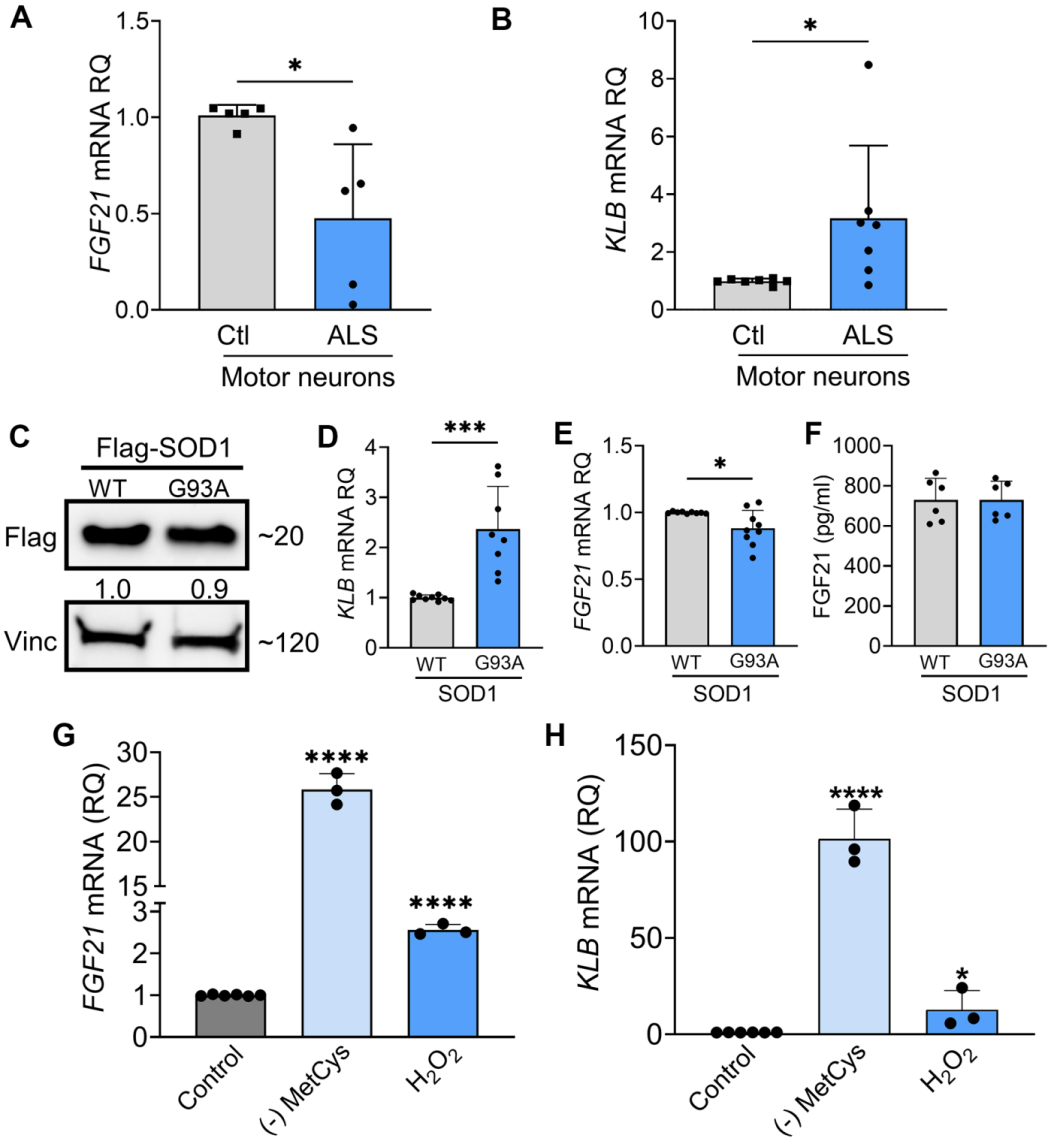
**FGF21-KLB signaling is dysregulated in ALS motor neurons.** (**A**) *FGF21* were measured in iPSC-derived motor neurons obtained from healthy controls and ALS patients carrying either C9orf72 or SOD1 mutations (Supplementary Table 1). *P = 0.012; two-tailed Mann Whitney test. (**B**) *KLB* mRNA levels were similarly measured in iPSC motor neurons. *P = 0.018; two-tailed Mann Whitney test. (**C**) NSC-34 motor neuron-like cells were transfected with FLAG-tagged WT and SOD1^G93A^ expression plasmids and lysates were assessed by western blot using the antibodies indicated. Bands were quantitated by densitometry and a ratio to the loading control, vinculin, was calculated (shown between the two blots). (**D**) *KLB* and (**E**) *FGF21* mRNA levels were measured in the same lysates. *P = 0.018, ***P = 0.0002, unpaired two-tailed t-test. (**F**) FGF21 protein was measured in the CM of transfected NSC-34 cells. (**G**) *FGF21* or (**H**) *KLB* mRNA levels were quantified from NSC-34 cells exposed to methionine-cystine (MetCys)-deprived media or treated with 100 mM H_2_O_2_ for 24 h. *P = 0.048, ****P < 0.0001; one-way ANOVA followed by Tukey’s multiple comparisons test. Data points represent biological replicates and bars are the mean ± SD.

As we found progressive loss of *KLB* expression in ALS spinal cord and muscle, we assessed the FGF21-KLB axis in motor neurons. Using iPSC-derived motor neurons from ALS patients (Supplementary Table 1), we assessed the expression of *FGF21* and *KLB* mRNAs. Compared to normal control iPSC motor neurons, there was a ~50% reduction in *FGF21* mRNA levels in ALS motor neuron samples (Figure 5A; *P =* 0.012). On the other hand, *KLB* mRNA was increased by 3-fold (Figure 5B; *P =* 0.018). We next transfected NSC-34 motor neuron-like cells with Flag-tagged wild-type (WT) SOD1 or SOD1^G93A^ expression cassettes (Figure 5C) and found a similar pattern of expression with a more than 2-fold increase in *KLB* (Figure 5D) and a ~15% reduction in *FGF21* in SOD1^G93A^-expressing cells versus WT (Figure 5E). FGF21 protein measured in the CM was unchanged (Figure 5F). As FGF21-KLB signaling is a major cellular stress response [16], we assessed the effect of oxidative stress on this pathway in NSC-34 cells. When cells were exposed to oxidative stress either by MetCys deprivation which leads to depletion of the intracellular antioxidant, glutathione [37, 38], or directly with H_2_O_2_ [39], there was a marked induction of *FGF21* and *KLB* mRNAs (Figure 5G, 5H). Induction of these genes was particularly pronounced with MetCys deprivation where *KLB* and *FGF21* mRNAs increased by nearly 100-fold and 25-fold respectively. The induction by H_2_O_2_ was less at two to three-fold. This response was mirrored by induction of stress-response genes, *ATF4* and *PGC-1α*, with MetCys deprivation showing the highest induction (Supplementary Figure 3A, 3B). *ATF4* was not induced by H_2_O_2_ in NSC-34 cells.

### FGF21 mitigates cytotoxic stress in G93A-SOD1-expressing NSC-34 cells and iPSC-derived ALS motor neurons

**Figure 6:**
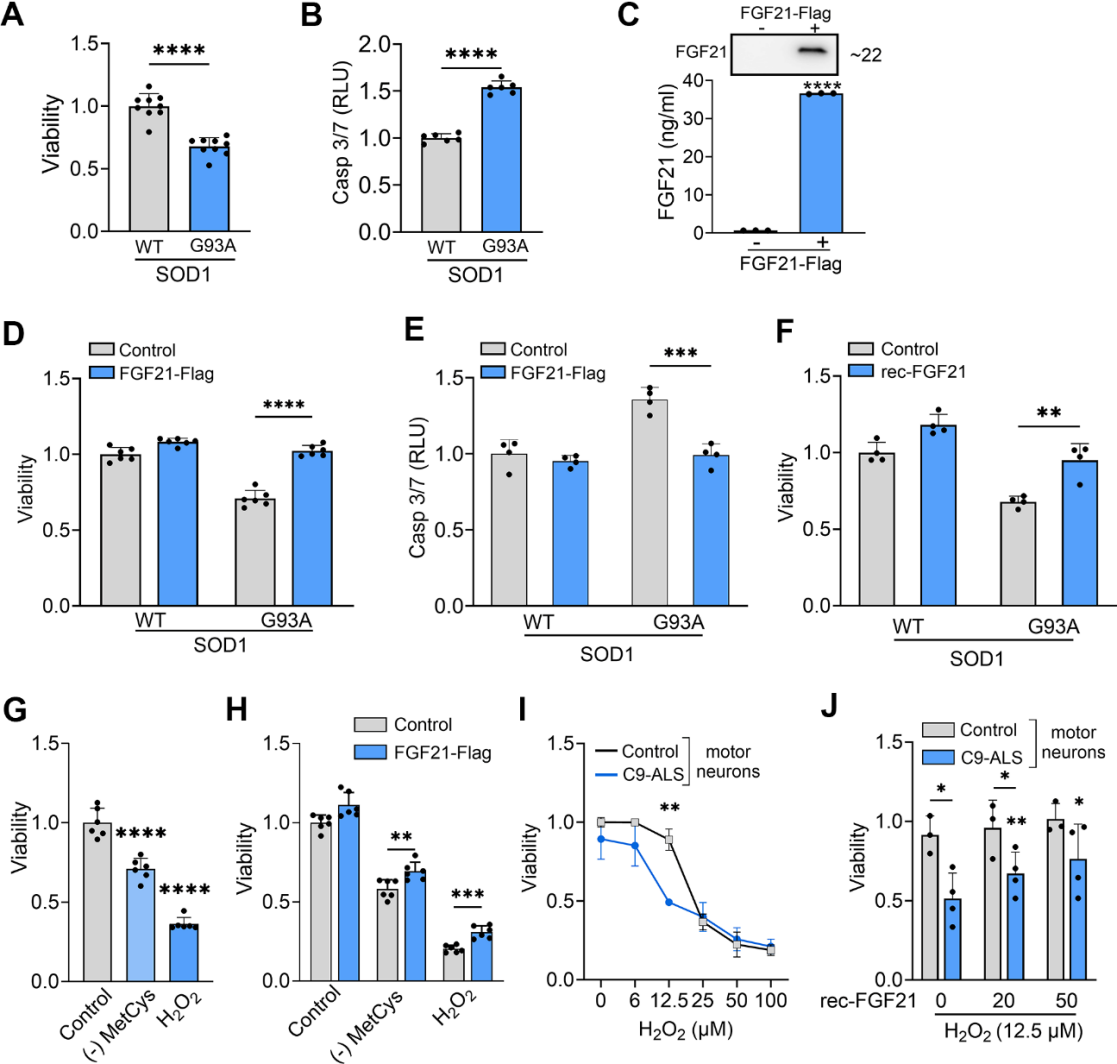
**FGF21 mitigates cytotoxicity in NSC-34 and ALS motor neurons induced oxidative stress.** (**A**) The viability of NSC-34 cells expressing FLAG-tagged WT-SOD1 or SOD1^G93A^ was determined using the Vialight assay. Viability for WT SOD1-transfected cells was set at 1. ****P < 0.0001, unpaired two-tailed t-test. (**B**) Caspase activation was measured in the same cells and values were normalized to activity in WT SOD1-transfected cells which was set at 1. ****P < 0.0001, unpaired two-tailed t-test. (**C**) FGF21 protein in the conditioned media of NSC-34 cells transfected with a FLAG-tagged FGF21 plasmid was detected by western blot (upper panel) and by ELISA (graph). ****P < 0.0001; unpaired two-tailed t-test. (**D**, **E**) NSC-34 cells expressing either WT-SOD1 or SOD1^G93A^ were transfected with FLAG-FGF21 and assessed for viability as in (**A**) and Caspase-3/7 as in (**B**). ****P < 0.0001, unpaired two-tailed t-test. (**F**) NSC-34 cells expressing either WT SOD1 or SOD1^G93A^ were treated with recombinant FGF21 (100 ng/ml) and assessed for viability as in (**A**). **P < 0.01, unpaired two-tailed t-test. (**G**) Cell viability was assessed in NSC-34 cells exposed to methionine-cystine (MetCys)-deprived media or treated with 100 mM H_2_O_2_. ****P < 0.0001; one-way ANOVA followed by Tukey’s multiple comparisons test. (**H**) NSC-34 cells transfected with FLAG-FGF21 (or empty vector) were subjected to stressors as described in (**G**) for 24h and then assayed for viability. **P = 0.007, ***P = 0.0002; unpaired two-tailed t-test. (**I**) iPSC motor neurons derived from C9-ALS patients or normal donors were exposed to different doses of H_2_O_2_ as indicated, and cell viability was assessed 24 h after treatment. **P < 0.01; unpaired two-tailed t-test with Welch’s correction. (**J**) iPSC motor neurons exposed to 12.5 μM of H_2_O_2,_ were treated with different doses (0, 25 and 50 ng/ml) of recombinant FGF21 and cell viability was assessed as in (**I**). *P < 0.05, **P < 0.01; paired two-tailed t-test among the different doses of rec-FGF21 treatment in C9-ALS groups and unpaired two-tailed test in normal vs. C9-ALS groups. Data points represent biological replicates and bars are the mean ± SD.

We first determined the impact of SOD1^G93A^ on cell viability and found a 33% reduction in viability (Figure 6A) and a concomitant 50% increase in Caspase-3/7 activity consistent with apoptotic activity (Figure 6B). Next, we ectopically expressed FGF21 in NSC-34 cells co-expressing wild-type (WT) SOD1 or SOD1^G93A^ to determine the effect on cell viability. We assayed the CM by ELISA and western blot and found a large increase in FGF21 in transfected cells at ~35 ng/ml (Figure 6C). Cell viability increased back to control levels along with reduced Caspase-3/7 activity (Figure 6D, 6E). In a separate experiment, we added recombinant FGF21 to the culture media of SOD1^G93A^-expressing NSC-34 cells and observed a similar reversal of cytotoxicity (Figure 6F). To assess further a potential paracrine effect of FGF21, NSC-34 cells expressing either (WT) SOD1 or SOD1^G93A^ and C2C12 cells expressing FGF21-Flag or EV were co-cultured in a transwell plate with a 3 μm porous filter (Supplementary Figure 4A). Viability was checked and found to be reduced in NSC-34 cells expressing SOD1^G93A^ co-cultured with C2C12-EV cells (*P* < 0.0001, Supplementary Figure 4B). When co-cultured with C2C12-FGF21 cells, loss of viability was blunted (*P* < 0.0001). We next exposed NSC-34 cells to MetCys deprivation or H_2_O_2_ and observed a significant loss of viability (*P* < 0.0001, Figure 6G) which was reversed with ectopic FGF21 (Figure 6H). We next treated human iPSC-derived motor neurons with different doses of H_2_O_2_ and found a difference in viability between control and ALS neurons, beginning at a dose of 6 μM and becoming significant at 12.5 μM (Figure 6I). We observed a concomitant and robust increase in *KLB* mRNA levels at 12.5 μM in ALS motor neurons compared to control (Supplementary Figure 5). Recombinant FGF21 treatment reversed this cytotoxicity in a dose-dependent manner (Figure 6J). In summary, SOD1^G93A^ or oxidative stress induced *FGF21* and *KLB* mRNA and triggered cytotoxicity in motor neurons which could be rescued by ectopic FGF21.

### FGF21 mitigates stress-induced toxicity in muscle cells and promotes myogenesis

**Figure 7:**
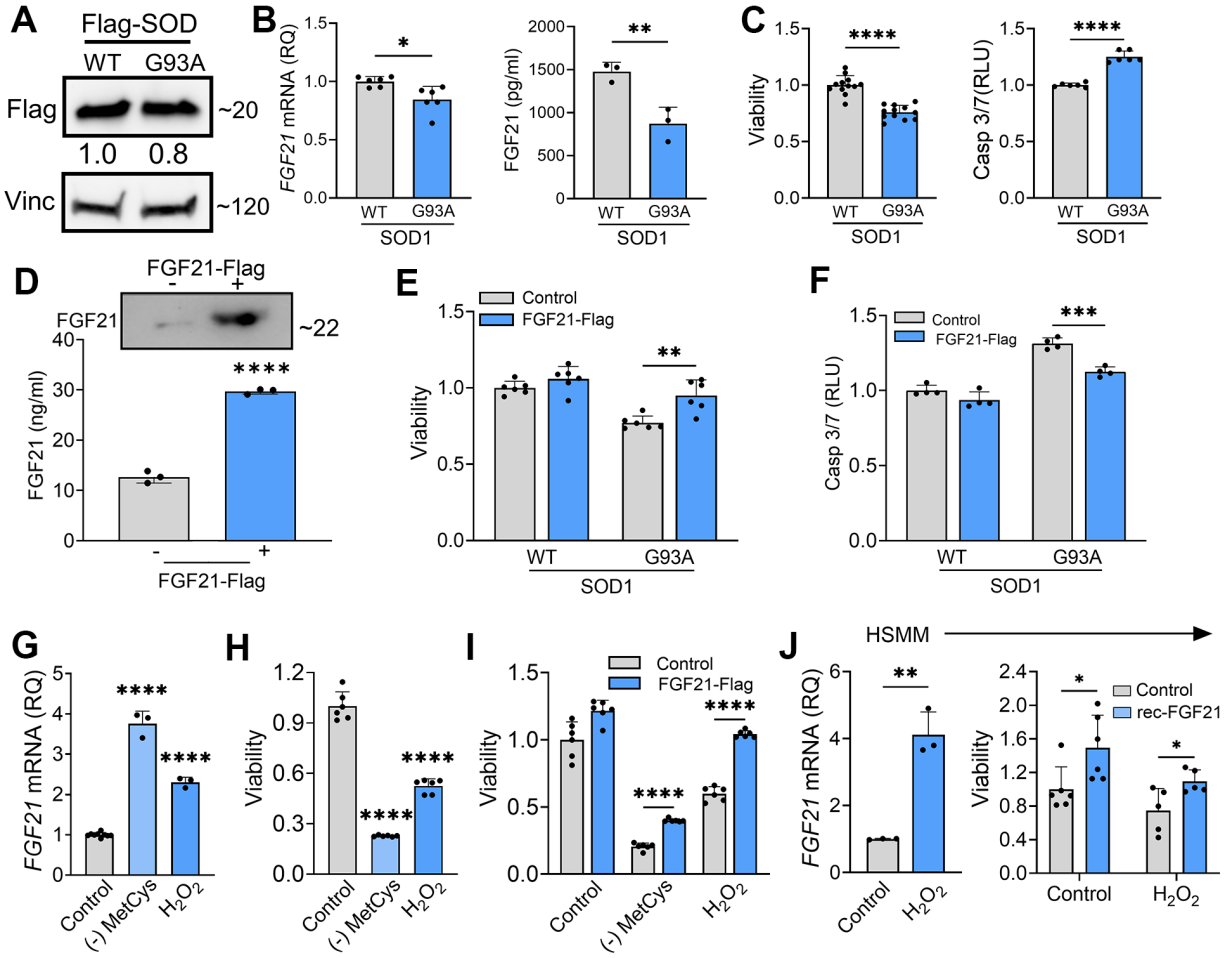
**FGF21 mitigates cytotoxicity in C2C12 and human primary myoblasts induced by oxidative stress.** (**A**) C2C12 myoblasts were transfected with FLAG-tagged WT and SOD1^G93A^ expression plasmids and lysates were assessed by western blot using the antibodies indicated. Bands were quantitated by densitometry and a ratio to the loading control, vinculin, was calculated (shown between the two blots). (**B**) *FGF21* mRNA levels were measured in the same lysates (left panel) and FGF21 protein in the conditioned media was quantified by ELISA (right panel). *P = 0.011, **P = 0.008; unpaired two-tailed t-test. Secretory FGF21 from the conditioned media was quantified using ELISA (right panel). (**C**) Viability of NSC-34 cells expressing FLAG-tagged WT-SOD1 or SOD1^G93A^ was determined using the Vialight assay (left panel). Caspase activation was measured in the same cells and values were normalized to activity in WT SOD1-transfected cells which was set at 1 (right panel). ****P < 0.0001, unpaired two-tailed t-test. (**D**) FGF21 protein in the conditioned media of C2C12 cells transfected with a FLAG-tagged FGF21 was detected by western blot (upper panel) and by ELISA (graph). Estimated size of the band (kDa) is shown to the right of the blot. ****P < 0.0001; unpaired two-tailed t-test. (**E**, **F**) C2C12 myoblasts cells expressing either WT-SOD1 or SOD1^G93A^ were transfected with FGF21-FLAG and assessed for viability and Caspase-3/7 activity as in (**C**). **P = 0.003, ***P = 0.0003; unpaired two-tailed t-test. (**G**) *FGF21* mRNA levels were quantified in C2C12 cells exposed to MetCys-deprived media or treated with 100 mM H_2_O_2_ for 24 h. ****P < 0.0001; one-way ANOVA followed by Tukey’s multiple comparisons test. (**H**) Cell viability was assessed in C2C12 myoblasts exposed to stressors as described in (**G**). ****P < 0.0001; one-way ANOVA followed by Tukey’s multiple comparisons test. (**I**) C2C12 cells transfected with FGF21-FLAG (or empty vector) were subjected to stressors as described in (**G**) for 24h and then assayed for viability. ****P < 0.0001; unpaired two-tailed t-test. (**J**) *FGF21* mRNA levels were quantified in human primary myoblast cells (HSMM) treated with 25 mM H_2_O_2_ for 24 h. **P = 0.0014; unpaired two-tailed t-test (left panel). HSMM cells treated with or without 25 mM H_2_O_2_ and recombinant FGF21 (100ng/ml) for 24h and then assayed for viability. *P < 0.05; unpaired two-tailed t-test (right panel). Data points represent biological replicates and bars are the mean ± SD.

Since FGF21 is known to exert autocrine effects [19], and we found that atrophic myofibers in ALS muscle express FGF21 (Figure 2), we next sought to assess the impact of FGF21 on muscle cells. Prior investigations have found that selective transgenic expression of SOD1^G93A^ in muscle induces oxidative stress leading to myofiber atrophy [40]. We transfected C2C12 cells with SOD1^G93A^ and found that FGF21 expression was significantly reduced compared to WT SOD1, with the CM showing a ~40% reduction in protein (Figure 7A, 7B). *KLB* mRNA was not detected, suggesting a potential β-klotho-independent function of FGF21. The pattern of FGF21 suppression resembled ALS motor neurons (Figure 5). With SOD1^G93A^ expression, there was a resultant loss of cell viability and an increase in Caspase-3/7 activity, each by 25%, compared to control (Figure 7C; *P <* 0.0001). We transfected the muscle cells with Flag-tagged FGF21 which resulted in a marked increase in protein expression detected in the CM (Figure 7D) and an attenuated loss of cell viability and apoptotic activity (Figure 7E, 7F). We next assessed the effect of oxidative stress and found an induction of *FGF21* with MetCys deprivation at 3.8-fold and H_2_O_2_ at 2.3-fold (Figure 7G; *P <* 0.0001). *ATF4* and *PGC-1α* were also induced with these stressors by 1.5 to 2.5-fold (Supplementary Figure 3C, 3D). Cell viability with MetCys deprivation led to a 75% reduction in viability and exposure to H_2_O_2_ resulted in a 50% reduction (Figure 7H). Ectopic FGF21 expression improved viability by 100% for MetCys deprivation and 73% for H_2_O_2_ (Figure 7I; *P <* 0.0001). In human primary myoblast cells there was a similar induction of *FGF21* at 4.1-fold when treated with H_2_O_2_ (Figure 7J, left panel; *P <* 0.01). The resultant loss of viability (~25%) was reversed by recombinant FGF21 treatment back to baseline levels (Figure 7J, right panel; *P <* 0.05). Taken together, oxidative stress induces FGF21 in C2C12 and human primary muscle cells, and the resultant cytotoxicity can be reversed by FGF21.

**Figure 8:**
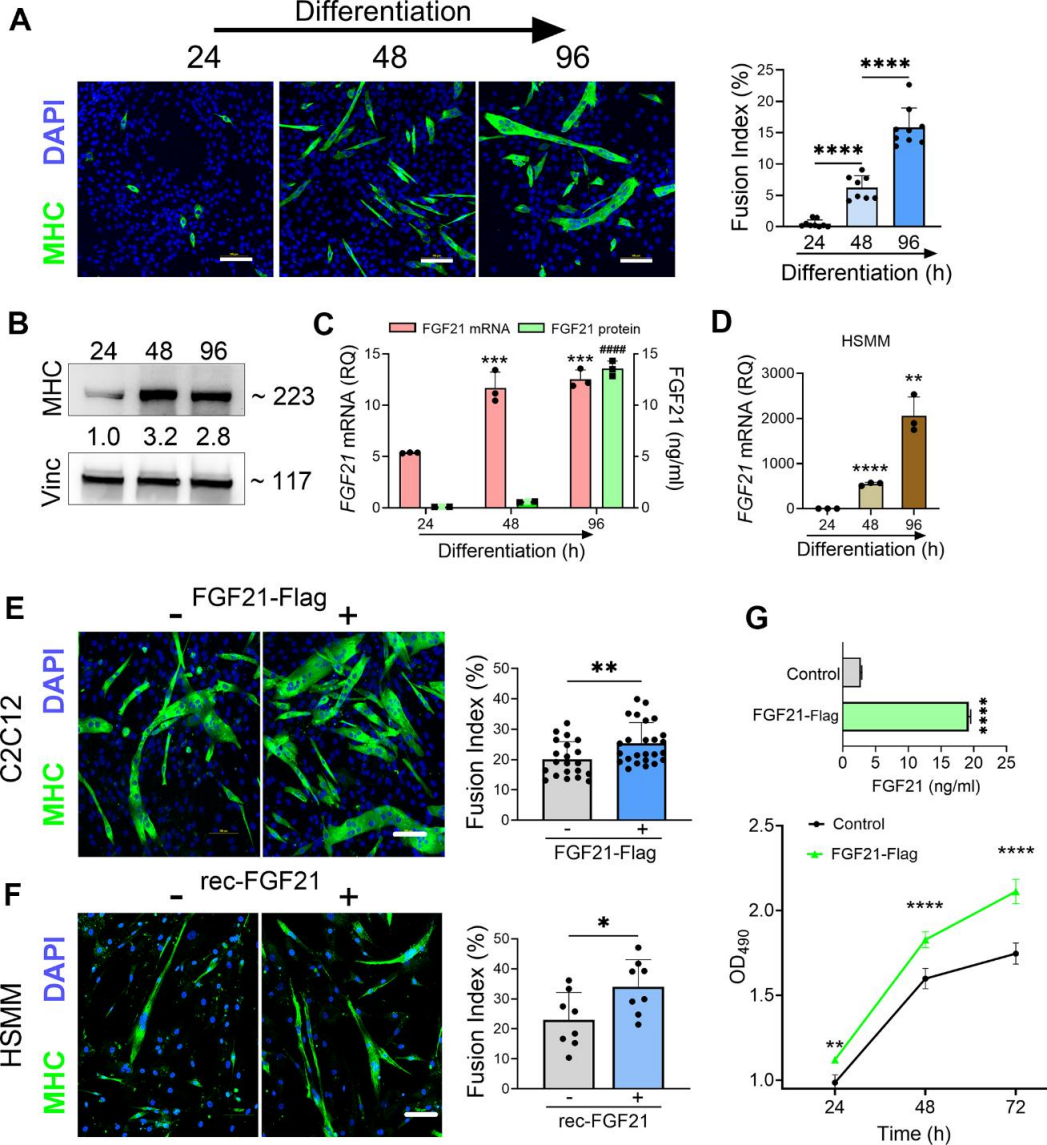
**FGF21 is upregulated during myogenesis and facilitates myogenic differentiation of C2C12 and human primary muscle cells (HSMM).** (**A**) C2C12 myoblasts were treated with DM for various time intervals and immunostained with an anti-MHC antibody followed by DAPI counterstaining. Myotube formation was detected by MHC-positive staining. Scale bars, 100 μm. The fusion index (%) was quantified as described in the methods (right panel). (**B**) C2C12 myoblasts treated with DM were lysed at specific time intervals and immunoblotted with antibodies against MHC and vinculin. Densitometry values (24 h time interval was set at 1) are shown. (**C**) *FGF21* mRNA levels were quantified from the lysates and FGF21 protein from conditioned media. ***P = 0.0005 comparing to the 24 h time interval, ^####^*P <* 0.0001 comparing to the 24 and 48 h time intervals; one-way ANOVA followed by Tukey’s multiple comparisons test. (**D**) HSMM cells were treated with DM for various time intervals and *FGF21* mRNA levels were quantified from the lysates as described in (**C**). **P < 0.01, ****P < 0.0001; unpaired two-tailed t-test. (**E**) C2C12 myoblasts were transfected with an FGF21-FLAG plasmid and cultured in DM for 96 h. Myotube formation was assessed by MHC-positive staining as in (**A**). Scale bar, 100 μm (left panel). Fusion index for transfected C2C12 cells. **P = 0.008; unpaired two-tailed t-test (right panel). (**F**) HSMM cells were treated with recombinant FGF21 (100ng/ml) and cultured in DM for 96 h. Myotube formation was assessed by MHC-positive staining as in (**A**, **E**). Scale bar, 100 μm (left panel). Fusion index for transfected HSMM cells. *P = 0.02; unpaired two-tailed t-test (right panel). (**G**) FGF21 levels in the conditioned media from C2C12 myoblasts transfected with FGF21-FLAG or empty vector control were quantified by ELISA (upper graph). ****P < 0.0001; unpaired two-tailed t test. Cells were reseeded and cultured in growth medium (GM) for 72 h. Cell proliferation was assessed at indicated time intervals using MTS (lower graph). **P = 0.007, ****P < 0.0001; one-way ANOVA followed by Tukey’s multiple comparisons test. Data points represent biological replicates and bars are the mean ± SD.

We further investigated the impact of FGF21 on myogenesis as prior studies in preclinical models of ALS have linked myogenesis to slower disease progression [41, 42]. We induced myogenic differentiation in C2C12 cells over 96 h resulting in an increased fusion index (Figure 8A) and induction of MHC (Figure 8B). Over the same time interval, there was a progressive and significant increase in both *FGF21* mRNA within cells and secreted FGF21 in the CM (Figure 8C). A similar time course of *FGF21* mRNA induction was observed with myogenic differentiation of human primary muscle cells, but the magnitude of induction was more robust (Figure 8D). Ectopic expression of FGF21in C2C12 cells increased myogenic differentiation as indicated by a higher fusion index (Figure 8E; *P =* 0.008) and a 2.2-fold induction of MHC (Supplementary Figure 6). Likewise myogenic differentiation in human primary muscle cells was increased with recombinant FGF21 treatment as reflected by an increased myotube fusion index (Figure 8F; *P* = 0.028). Cell proliferation was then measured in C2C12 cells transfected with FGF21-FLAG (Figure 8G). Just prior to seeding, FGF21 was quantitated in the CM by ELISA and showed a ~7-fold increase in FGF21 in the FGF21-FLAG transfected cells versus empty vector control. We observed a significant increase in proliferation in FGF21-FLAG transfected cells at all time intervals tested, reaching 25% at 72 h (*P <* 0.0001). In summary, FGF21 induction occurs as part of the myogenic differentiation program, where it appears to play a positive role in myogenesis.

## DISCUSSION

In this study, we found that FGF21 is prominently upregulated in atrophic myofibers in patients with ALS and increases with disease progression. FGF21 is also increased in plasma of ALS patients as a group, but disproportionately in those with slower clinical progression and higher BMI. We show that RNA expression of the key co-receptor, β-Klotho, is upregulated in ALS motor neurons at baseline and that both *FGF21* and *KLB* are induced by oxidative stress with FGF21 providing a protective effect. In muscle cells, FGF21 promotes myogenesis and exerts a similar protective effect against cytotoxic stress. Taken together, these findings identify FGF21 as a biomarker that provides insight into the clinical heterogeneity and pathophysiology of ALS while at the same time identifying it as a potential target for future therapeutic development.

Although liver is the predominant source of FGF21 in physiological states, mitochondrial dysfunction, as seen in skeletal muscle at the earliest stages of ALS, is a potent stimulus for FGF21 induction [16, 43, 44]. In mitochondrial myopathies, elevated serum FGF21 from skeletal muscle is now recognized as a diagnostic biomarker [45–47]. Our data suggest that one source of elevated FGF21 in plasma is from skeletal muscle as the protein levels were high (more than 2-fold higher than plasma levels and 20-fold higher than spinal cord tissue) and immunohistochemistry showed substantial extravasation into the endomysial space (Figures 1–3). Unlike most FGF family members, FGF21 has a lower affinity for heparan sulfate glycosaminoglycans [48] which are present in the basal lamina surrounding muscle fibers [49], and thus less likely to get sequestered. This is in contrast to FGF23, a related family member that we previously identified in ALS muscle samples but could not detect in plasma samples [24]. Under physiological conditions, liver is the predominant source of circulating FGF21 [20, 36]. Our findings in the SOD1^G93A^ mouse, however, suggest that muscle is a major source as there was a ~2-fold higher expression level versus liver (Supplementary Figure 2). Interestingly, we found increased FGF21 protein in spinal cord, but there was no overall increase in *FGF21* mRNA, raising the possibility that FGF21 may be coming from the periphery. FGF21 can readily cross the blood-brain barrier, and elevated plasma levels have previously been shown to correlate with elevated cerebrospinal fluid (CSF) levels in humans [50, 51]. On the other hand, we detected increase *FGF21* transcripts in whole spinal cord of the SOD1^G93A^ mouse at a very early, presymptomatic, stage (~ 6 weeks; Figure 1). Others have detected induction of *FGF21* in a subset motor neurons in the SOD1^G93A^ and TDP43 mouse models in late presymptomatic/early symptomatic stages (11-12 weeks post natal) [52, 53]. Although protein levels were not measured, one of the studies found a high association of *FGF21* with ribosomes in motor neurons [53]. It is possible that post-transcriptional/translational mechanisms are at play within the CNS, particularly since the protein half-life of FGF21 is short [36]. Of note, FGF21 has been proposed as a potential early CNS biomarker in other animal models of neurodegenerative disease [54].

The potential for FGF21 to track disease progression is supported by several lines of evidence. First, since a major hallmark of ALS is progressive muscle atrophy [33], FGF21 would be expected to increase over time because of its correlation with atrophied myofibers (Figure 2) and HDAC4 (Supplementary Figure 1), a muscle biomarker that associates with progressive muscle denervation in ALS [31]. Indeed, this is supported by the nearly 8-fold increase in muscle *FGF21* mRNA levels in end-stage ALS muscle versus muscle biopsy samples at earlier stages (Figure 1). There was also a progressive increase in *FGF21* mRNA in ALS mouse muscle starting in the presymptomatic stages (Figure 1). If muscle FGF21 were the only source for increased plasma FGF21, however, higher levels might be expected to associate with more advanced disease (i.e. more muscle atrophy) as has been observed with mitochondrial myopathies [46, 55]. In our longitudinal study of 16 patients, however, those with high FGF21 plasma levels showed slower disease progression and longer survival (Figure 3). This raises the possibility that in some ALS patients, other sources contribute to circulating FGF21 such as liver. A notable feature of the subset with high circulating FGF21 levels was a higher mean BMI which verged on obesity. Prior studies have shown a strong positive correlation between serum FGF21 levels and BMI, particularly increased fat mass, with liver being the likely source [50, 56]. Increased BMI has consistently been linked to longer survival in ALS in a number of clinical studies [57–60]. In the Pooled Resource Open-Access ALS Clinical Trials database (PRO-ACT), patients with a BMI between 25 – 30 kg/m^2^ (similar to the mean value of 29.2 kg/m^2^ in the high FGF21 subset reported here) had a 35% reduced risk of dying compared to patients with BMI < 25 kg/m^2^ (the low FGF21 group in our study had a mean BMI of 22.6 kg/m^2^) [59]. In a pilot study of ALS patients, we previously found that low percentage body fat and loss of total fat mass correlated with faster monthly declines in ALSFRS-R scores [61]. In another study, patients with low visceral fat mass at baseline had significantly faster disease progression [62]. The majority of patients in our study with low circulating FGF21 levels had bulbar onset ALS (Supplementary Table 4) which is associated with shorter lifespan and weight loss prior to diagnosis (likely due to decreased nutritional intake) [63, 64]. These low levels may also reflect a possible impairment in FGF21 adaptive responses as observed in other age-associated diseases including Alzheimer’s and type II diabetes [21].

A limitation of our plasma FGF21 study is the small number of patients, which in a heterogeneous disorder such as ALS, can lead to premature conclusions. A larger ALS cohort will be required for validation. The findings are timely, however, given the increasing attention to dysregulation of energy metabolism as a key driver of ALS disease progression [6, 65–68]. FGF21 is a master regulator of metabolic and nutrient homeostasis and a key hormone in adapting to states of energy deprivation [36]. While it is well established that FGF21 is a key regulator of fatty acid oxidation in the liver [19], less is known about this function in skeletal muscle or in the CNS [20]. Preclinical studies in the ALS mouse suggest that fatty acid oxidation becomes a major source of energy in skeletal muscle in the SOD1^G93A^ [69–72]. In myotubes derived from human ALS muscle, increased fatty acid oxidation capacity was associated with slower disease progression [70]. It is interesting to note that a recent study on macronutrients in ALS suggests that a high glycemic index diet, which is a potent stimulus for FGF21 induction by the liver [73], is linked to slower disease progression in patients with ALS [74].

The biology of FGF21 is complex because of its broad and divergent effects on different organs that vary depending on the physiological or pathological context [19, 36]. In our study, we found increased FGF21 in skeletal muscle, spinal cord and in the circulation, suggesting that it could impact ALS pathophysiology, including stress response, autophagy, energy metabolism, and inflammation, at multiple levels. Because of the increase in FGF21 in the CNS (Figure 1) and the previously reported correlation between higher CSF FGF21 levels and high serum FGF21 (and concomitant BMI) [50], potential target cell populations affected in ALS are also broad. This impact is underscored by preclinical studies in Parkinson’s, Alzheimer’s and other CNS disease where FGF21 promotes neuroprotection and mitigates neurodegeneration through effects on different cell populations which crossover to ALS [75–81]. This includes suppression of glial activation and neuroinflammation, glutamate excitotoxicity, reversal of defective astrocyte-neuron lactate shuttling, increased neuronal survival, and enhanced myelin regeneration mediated by oligodendrocytes. We focused on the potential trophic effects of FGF21 based on prior studies showing a direct protective effect of FGF family members, FGF1 and 2, on motor neurons after spinal cord injury [82, 83]. A possible benefit of FGF21 signaling was also suggested in a prior study with R1Mab1, an FGFR1-targeting antibody that activates the receptor, in the SOD1^G93A^ mouse where there was mild amelioration of motor phenotype [84].

A key component of FGF21 signaling is the co-receptor, β-klotho (KLB), which exerts cellular specificity of signaling as the FGF receptors are nearly ubiquitous [36]. Our detection of *KLB* in iPSC-derived human motor neurons (Figure 5) indicates a high likelihood that FGF21 signaling is relevant to motor neuron physiology. Evidence for FGF21 signaling as a stress response in motor neurons was further supported by our finding of *KLB* and *FGF21* induction in NSC-34 cells with oxidative stress, either by exposure to H_2_O_2_ or deprivation of MetCys (Figure 5). Interestingly, we found more than a 3-fold increase in *KLB* mRNA in ALS motor neurons and this pattern was recapitulated in NSC-34 motor neuron-like cells expressing SOD1^G93A^ (Figure 5). This baseline increase suggests that the trigger for FGF signaling is already present in ALS motor neurons at the time of differentiation. Oxidative stress has previously been observed in differentiated motor neurons from ALS patients using intracellular biosensors, and this might reflect an early and intrinsic vulnerability of ALS motor neurons [85, 86]. At the same time, however, basal *FGF21* levels were reduced, suggesting an impairment in the FGF21-KLB axis. Increased levels of *KLB* in motor neurons from ALS patients in response to oxidative stress further validate our hypothesis of an impaired FGF21-KLB axis (Supplementary Figure 5). In ALS tissue samples, we observed an upregulation of *KLB* in muscle biopsies but a downregulation in end-stage muscle and spinal cord (Figure 4). This pattern could be consistent with disease-associated loss of motor neurons. Of note, we were unable to detect *KLB* mRNA expression in human primary muscle cells (or C2C12 cells) even after exposure to H_2_O_2_. KLB has been detected in muscle at very low levels [20], but the source could be from other cell types in muscle tissue including motor neurons. We cannot exclude the possibility that loss of myofibers contributed to reduced *KLB* expression observed in end-stage muscle. Nonetheless, the concomitant higher FGF21 levels in ALS muscle and spinal cord at end-stage suggest an ongoing adaptive response to disease progression. The trophic effect of FGF21 on SOD1^G93A^ and C9-ALS motor neurons was supported by the reversal of cytotoxicity and apoptosis with ectopic expression (Figure 6). Potential mechanisms for this rescue effect include attenuation of oxidative stress, modulation of autophagy, improved energy metabolism and mitochondrial function [76, 77].

Selective transgenic expression of SOD1^G93A^ in skeletal muscle has been shown to promote mitochondrial dysfunction, oxidative stress, and progressive atrophy [40]. We found a similar toxic effect in muscle cells and that ectopic FGF21 expression reversed this toxicity (Figure 7). FGF21 activates mTOR signaling in muscle cells leading to improved mitochondrial function, energy metabolism and reversal of mitochondrial impairment by oxidative stress [87]. In addition to a rescue effect, we also found that FGF21 promoted myogenesis, including enhanced proliferation and differentiation of C2C12 myoblasts and in human primary myoblasts (Figure 8), as observed by other investigators [88, 89]. A prior study assessing satellite cells from human ALS muscle tissue revealed defects in myogenesis, particularly differentiation and maturation [90]. Defects in myogenesis were also observed in satellite cells derived from SOD1^G93A^ mice at early pre-symptomatic stages [91] and in C2C12 cells expressing SOD1^G93A^ [92]. Increased myogenesis was identified as a key mechanism for the slow-progressing phenotype in SOD1^G93A^ mice from the C57 background [41, 42]. One study found that FGF21 drives the molecular transition of myoblasts to an aerobic (slow twitch fiber-like) phenotype [88], a process that occurs in ALS muscle due to the vulnerability and early degeneration of motor neurons innervating fast-twitch (Type II) muscle fibers [93, 94].

FGF21 has been identified as a biomarker of frailty because of its association with age-related metabolic disorders [23, 95, 96]. Aging is a key risk factor for ALS, and many pathophysiological features of ALS overlap with normal aging patterns including mitochondrial dysfunction, oxidative stress and inflammation [97]. The role of FGF21 signaling in these age-associated processes is unclear. Many studies support a mitigating effect of FGF21 on aging, age-related diseases, mitochondrial function, muscle atrophy and function via its effects on metabolism [21, 23, 95]. Other studies suggest FGF21 signaling exerts a negative effect on myogenesis and promotes atrophy in different physiological and disease contexts including fasting, aging, intrauterine growth restriction, and short-term pharmacological administration [98–101]. A limitation of many studies investigating FGF21 *in vivo*, including ours, is that the immunoassays for detection do not distinguish between active (uncleaved) and inactive (cleaved) forms of FGF21 [102], thus underscoring the complexity of FGF21 biology.

In summary, we have identified FGF21 as a novel biomarker in ALS that is detected in multiple compartments including muscle, spinal cord, and circulation. It is strongly expressed in atrophied myofibers, and high plasma levels associated with slower disease progression. For the first time, we show that the FGF21-KLB axis is a relevant stress response pathway in motor neurons and that it mitigates cytotoxicity induced by oxidative stress and co-expression of mutant SOD1. It has a similar protective effect in muscle cells where it also promotes myogenesis. Future studies will need to test FGF21 using *in vivo* models of ALS, including tissue-specific or systemic delivery, to validate the mitigating effects it appears to have *in cellulo* and to assess its potential as a novel therapy in ALS. In the clinic, plasma FGF21 levels might have value as a prognostic biomarker but will need validation in larger multi-center studies.

## Supplementary Material

Supplementary Figures

Supplementary Tables

## References

[1] Kazamel M, Cutter G, Claussen G, Alsharabati M, Oh SJ, Lu L, King PH. Epidemiological features of amyotrophic lateral sclerosis in a large clinic-based African American population. Amyotroph Lateral Scler Frontotemporal Degener. 2013; 14:334–7. 10.3109/21678421.2013.770030 23458155 PMC3732496

[2] Hardiman O, van den Berg LH, Kiernan MC. Clinical diagnosis and management of amyotrophic lateral sclerosis. Nat Rev Neurol. 2011; 7:639–49. 10.1038/nrneurol.2011.153 21989247

[3] Verma S, Khurana S, Vats A, Sahu B, Ganguly NK, Chakraborti P, Gourie-Devi M, Taneja V. Neuromuscular Junction Dysfunction in Amyotrophic Lateral Sclerosis. Mol Neurobiol. 2022; 59:1502–27. 10.1007/s12035-021-02658-6 34997540

[4] King PH. Skeletal muscle as a molecular and cellular biomarker of disease progression in amyotrophic lateral sclerosis: a narrative review. Neural Regen Res. 2024; 19:747–53. 10.4103/1673-5374.382226 37843208 PMC10664124

[5] Moloney EB, de Winter F, Verhaagen J. ALS as a distal axonopathy: molecular mechanisms affecting neuromuscular junction stability in the presymptomatic stages of the disease. Front Neurosci. 2014; 8:252. 10.3389/fnins.2014.00252 25177267 PMC4132373

[6] Nelson AT, Trotti D. Altered Bioenergetics and Metabolic Homeostasis in Amyotrophic Lateral Sclerosis. Neurotherapeutics. 2022; 19:1102–18. 10.1007/s13311-022-01262-3 35773551 PMC9587161

[7] Kwan T, Kazamel M, Thoenes K, Si Y, Jiang N, King PH. Wnt antagonist FRZB is a muscle biomarker of denervation atrophy in amyotrophic lateral sclerosis. Sci Rep. 2020; 10:16679. 10.1038/s41598-020-73845-z 33028902 PMC7541525

[8] Si Y, Cui X, Kim S, Wians R, Sorge R, Oh SJ, Kwan T, AlSharabati M, Lu L, Claussen G, Anderson T, Yu S, Morgan D, et al. Smads as muscle biomarkers in amyotrophic lateral sclerosis. Ann Clin Transl Neurol. 2014; 1:778–87. 10.1002/acn3.117 25493269 PMC4241805

[9] Si Y, Kazamel M, Kwon Y, Lee I, Anderson T, Zhou S, Bamman M, Wiggins D, Kwan T, King PH. The vitamin D activator CYP27B1 is upregulated in muscle fibers in denervating disease and can track progression in amyotrophic lateral sclerosis. J Steroid Biochem Mol Biol. 2020; 200:105650. 10.1016/j.jsbmb.2020.105650 32142934 PMC7274892

[10] Si Y, Kim S, Cui X, Zheng L, Oh SJ, Anderson T, AlSharabati M, Kazamel M, Volpicelli-Daley L, Bamman MM, Yu S, King PH. Transforming Growth Factor Beta (TGF-β) Is a Muscle Biomarker of Disease Progression in ALS and Correlates with Smad Expression. PLoS One. 2015; 10:e0138425. 10.1371/journal.pone.0138425 26375954 PMC4574401

[11] Tsitsipatis D, Mazan-Mamczarz K, Si Y, Herman AB, Yang JH, Guha A, Piao Y, Fan J, Martindale JL, Munk R, Yang X, De S, Singh BK, et al. Transcriptomic analysis of human ALS skeletal muscle reveals a disease-specific pattern of dysregulated circRNAs. Aging (Albany NY). 2022; 14:9832–59. 10.18632/aging.204450 36585921 PMC9831722

[12] Si Y, Cui X, Crossman DK, Hao J, Kazamel M, Kwon Y, King PH. Muscle microRNA signatures as biomarkers of disease progression in amyotrophic lateral sclerosis. Neurobiol Dis. 2018; 114:85–94. 10.1016/j.nbd.2018.02.009 29486297 PMC5891369

[13] Lopez MA, Si Y, Hu X, Williams V, Qushair F, Carlyle J, Alesce L, Conklin M, Gilbert S, Bamman MM, Alexander MS, King PH. Smad8 Is Increased in Duchenne Muscular Dystrophy and Suppresses miR-1, miR-133a, and miR-133b. Int J Mol Sci. 2022; 23:7515. 10.3390/ijms23147515 35886863 PMC9323105

[14] Darabid H, Perez-Gonzalez AP, Robitaille R. Neuromuscular synaptogenesis: coordinating partners with multiple functions. Nat Rev Neurosci. 2014; 15:703–18. 25493308

[15] Benatar M, Boylan K, Jeromin A, Rutkove SB, Berry J, Atassi N, Bruijn L. ALS biomarkers for therapy development: State of the field and future directions. Muscle Nerve. 2016; 53:169–82. 10.1002/mus.24979 26574709 PMC4718795

[16] Tezze C, Romanello V, Sandri M. FGF21 as Modulator of Metabolism in Health and Disease. Front Physiol. 2019; 10:419. 10.3389/fphys.2019.00419 31057418 PMC6478891

[17] Jena J, García-Peña LM, Pereira RO. The roles of FGF21 and GDF15 in mediating the mitochondrial integrated stress response. Front Endocrinol (Lausanne). 2023; 14:1264530. 10.3389/fendo.2023.1264530 37818094 PMC10561105

[18] Yan B, Mei Z, Tang Y, Song H, Wu H, Jing Q, Zhang X, Yan C, Han Y. FGF21-FGFR1 controls mitochondrial homeostasis in cardiomyocytes by modulating the degradation of OPA1. Cell Death Dis. 2023; 14:311. 10.1038/s41419-023-05842-9 37156793 PMC10167257

[19] Fisher FM, Maratos-Flier E. Understanding the Physiology of FGF21. Annu Rev Physiol. 2016; 78:223–41. 10.1146/annurev-physiol-021115-105339 26654352

[20] Sun H, Sherrier M, Li H. Skeletal Muscle and Bone - Emerging Targets of Fibroblast Growth Factor-21. Front Physiol. 2021; 12:625287. 10.3389/fphys.2021.625287 33762965 PMC7982600

[21] Conte M, Sabbatinelli J, Chiariello A, Martucci M, Santoro A, Monti D, Arcaro M, Galimberti D, Scarpini E, Bonfigli AR, Giuliani A, Olivieri F, Franceschi C, Salvioli S. Disease-specific plasma levels of mitokines FGF21, GDF15, and Humanin in type II diabetes and Alzheimer’s disease in comparison with healthy aging. Geroscience. 2021; 43:985–1001. 10.1007/s11357-020-00287-w 33131010 PMC8110619

[22] Hill CM, Albarado DC, Coco LG, Spann RA, Khan MS, Qualls-Creekmore E, Burk DH, Burke SJ, Collier JJ, Yu S, McDougal DH, Berthoud HR, Münzberg H, et al. FGF21 is required for protein restriction to extend lifespan and improve metabolic health in male mice. Nat Commun. 2022; 13:1897. 10.1038/s41467-022-29499-8 35393401 PMC8991228

[23] Mishra M, Wu J, Kane AE, Howlett SE. The intersection of frailty and metabolism. Cell Metab. 2024; 36:893–911. 10.1016/j.cmet.2024.03.012 38614092 PMC11123589

[24] Si Y, Kazamel M, Benatar M, Wuu J, Kwon Y, Kwan T, Jiang N, Kentrup D, Faul C, Alesce L, King PH. FGF23, a novel muscle biomarker detected in the early stages of ALS. Sci Rep. 2021; 11:12062. 10.1038/s41598-021-91496-6 34103575 PMC8187665

[25] Lu L, Zheng L, Viera L, Suswam E, Li Y, Li X, Estévez AG, King PH. Mutant Cu/Zn-superoxide dismutase associated with amyotrophic lateral sclerosis destabilizes vascular endothelial growth factor mRNA and downregulates its expression. J Neurosci. 2007; 27:7929–38. 10.1523/JNEUROSCI.1877-07.2007 17652584 PMC6672720

[26] Selvaraj BT, Livesey MR, Zhao C, Gregory JM, James OT, Cleary EM, Chouhan AK, Gane AB, Perkins EM, Dando O, Lillico SG, Lee YB, Nishimura AL, et al. C9ORF72 repeat expansion causes vulnerability of motor neurons to Ca^2+^-permeable AMPA receptor-mediated excitotoxicity. Nat Commun. 2018; 9:347. 10.1038/s41467-017-02729-0 29367641 PMC5783946

[27] Yang S, Wijegunawardana D, Sheth U, Veire AM, Salgado JMS, Agrawal M, Zhou J, Pereira JD, Gendron TF, Guo JU. Aberrant splicing exonizes *C9ORF72* repeat expansion in ALS/FTD. bioRxiv [Preprint]. 2023; 2023.11.13.566896. 10.1101/2023.11.13.566896 40790269 PMC12720984

[28] Lu L, Wang S, Zheng L, Li X, Suswam EA, Zhang X, Wheeler CG, Nabors LB, Filippova N, King PH. Amyotrophic lateral sclerosis-linked mutant SOD1 sequesters Hu antigen R (HuR) and TIA-1-related protein (TIAR): implications for impaired post-transcriptional regulation of vascular endothelial growth factor. J Biol Chem. 2009; 284:33989–98. 10.1074/jbc.M109.067918 19805546 PMC2797169

[29] Noë S, Corvelyn M, Willems S, Costamagna D, Aerts JM, Van Campenhout A, Desloovere K. The Myotube Analyzer: how to assess myogenic features in muscle stem cells. Skelet Muscle. 2022; 12:12. 10.1186/s13395-022-00297-6 35689270 PMC9185954

[30] Logroscino G, Traynor BJ, Hardiman O, Chiò A, Mitchell D, Swingler RJ, Millul A, Benn E, Beghi E, and EURALS. Incidence of amyotrophic lateral sclerosis in Europe. J Neurol Neurosurg Psychiatry. 2010; 81:385–90. 10.1136/jnnp.2009.183525 19710046 PMC2850819

[31] Bruneteau G, Simonet T, Bauché S, Mandjee N, Malfatti E, Girard E, Tanguy ML, Behin A, Khiami F, Sariali E, Hell-Remy C, Salachas F, Pradat PF, et al. Muscle histone deacetylase 4 upregulation in amyotrophic lateral sclerosis: potential role in reinnervation ability and disease progression. Brain. 2013; 136:2359–68. 10.1093/brain/awt164 23824486

[32] Vinsant S, Mansfield C, Jimenez-Moreno R, Del Gaizo Moore V, Yoshikawa M, Hampton TG, Prevette D, Caress J, Oppenheim RW, Milligan C. Characterization of early pathogenesis in the SOD1(G93A) mouse model of ALS: part II, results and discussion. Brain Behav. 2013; 3:431–57. 10.1002/brb3.142 24381813 PMC3869683

[33] Al-Sarraj S, King A, Cleveland M, Pradat PF, Corse A, Rothstein JD, Leigh PN, Abila B, Bates S, Wurthner J, Meininger V. Mitochondrial abnormalities and low grade inflammation are present in the skeletal muscle of a minority of patients with amyotrophic lateral sclerosis; an observational myopathology study. Acta Neuropathol Commun. 2014; 2:165. 10.1186/s40478-014-0165-z 25510661 PMC4297389

[34] Post A, Dam WA, Sokooti S, Groothof D, Gloerich J, van Gool AJ, Kremer D, Gansevoort RT, van den Born J, Kema IP, Franssen CF, Dullaart RP, Bakker SJ. Circulating FGF21 Concentration, Fasting Plasma Glucose, and the Risk of Type 2 Diabetes: Results From the PREVEND Study. J Clin Endocrinol Metab. 2023; 108:1387–93. 10.1210/clinem/dgac729 36533509 PMC10188302

[35] Labra J, Menon P, Byth K, Morrison S, Vucic S. Rate of disease progression: a prognostic biomarker in ALS. J Neurol Neurosurg Psychiatry. 2016; 87:628–32. 10.1136/jnnp-2015-310998 26152368

[36] BonDurant LD, Potthoff MJ. Fibroblast Growth Factor 21: A Versatile Regulator of Metabolic Homeostasis. Annu Rev Nutr. 2018; 38:173–96. 10.1146/annurev-nutr-071816-064800 29727594 PMC6964258

[37] Paul BD, Sbodio JI, Snyder SH. Cysteine Metabolism in Neuronal Redox Homeostasis. Trends in Pharmacological Sciences. 2018; 39:513–24. 10.1016/j.tips.2018.02.007 29530337 PMC5912966

[38] Upadhyayula PS, Higgins DM, Mela A, Banu M, Dovas A, Zandkarimi F, Patel P, Mahajan A, Humala N, Nguyen TTT, Chaudhary KR, Liao L, Argenziano M, et al. Dietary restriction of cysteine and methionine sensitizes gliomas to ferroptosis and induces alterations in energetic metabolism. Nat Commun. 2023; 14:1187. 10.1038/s41467-023-36630-w 36864031 PMC9981683

[39] Gille JJ, Joenje H. Cell culture models for oxidative stress: superoxide and hydrogen peroxide versus normobaric hyperoxia. Mutat Res. 1992; 275:405–14. 10.1016/0921-8734(92)90043-o 1383781

[40] Dobrowolny G, Aucello M, Rizzuto E, Beccafico S, Mammucari C, Boncompagni S, Belia S, Wannenes F, Nicoletti C, Del Prete Z, Rosenthal N, Molinaro M, Protasi F, et al. Skeletal muscle is a primary target of SOD1G93A-mediated toxicity. Cell Metab. 2008; 8:425–36. 10.1016/j.cmet.2008.09.002 19046573

[41] Margotta C, Fabbrizio P, Ceccanti M, Cambieri C, Ruffolo G, D’Agostino J, Trolese MC, Cifelli P, Alfano V, Laurini C, Scaricamazza S, Ferri A, Sorarù G, et al. Immune-mediated myogenesis and acetylcholine receptor clustering promote a slow disease progression in ALS mouse models. Inflamm Regen. 2023; 43:19. 10.1186/s41232-023-00270-w 36895050 PMC9996869

[42] Trolese MC, Scarpa C, Melfi V, Fabbrizio P, Sironi F, Rossi M, Bendotti C, Nardo G. Boosting the peripheral immune response in the skeletal muscles improved motor function in ALS transgenic mice. Mol Ther. 2022; 30:2760–84. 10.1016/j.ymthe.2022.04.018 35477657 PMC9372324

[43] Loeffler JP, Picchiarelli G, Dupuis L, Gonzalez De Aguilar JL. The Role of Skeletal Muscle in Amyotrophic Lateral Sclerosis. Brain Pathol. 2016; 26:227–36. 10.1111/bpa.12350 26780251 PMC8029271

[44] Zhou J, Yi J, Fu R, Liu E, Siddique T, Ríos E, Deng HX. Hyperactive intracellular calcium signaling associated with localized mitochondrial defects in skeletal muscle of an animal model of amyotrophic lateral sclerosis. J Biol Chem. 2010; 285:705–12. 10.1074/jbc.M109.041319 19889637 PMC2804218

[45] Suomalainen A, Elo JM, Pietiläinen KH, Hakonen AH, Sevastianova K, Korpela M, Isohanni P, Marjavaara SK, Tyni T, Kiuru-Enari S, Pihko H, Darin N, Õunap K, et al. FGF-21 as a biomarker for muscle-manifesting mitochondrial respiratory chain deficiencies: a diagnostic study. Lancet Neurol. 2011; 10:806–18. 10.1016/S1474-4422(11)70155-7 21820356 PMC7568343

[46] Lehtonen JM, Forsström S, Bottani E, Viscomi C, Baris OR, Isoniemi H, Höckerstedt K, Österlund P, Hurme M, Jylhävä J, Leppä S, Markkula R, Heliö T, et al. FGF21 is a biomarker for mitochondrial translation and mtDNA maintenance disorders. Neurology. 2016; 87:2290–9. 10.1212/WNL.0000000000003374 27794108 PMC5270510

[47] Tyynismaa H, Carroll CJ, Raimundo N, Ahola-Erkkilä S, Wenz T, Ruhanen H, Guse K, Hemminki A, Peltola-Mjøsund KE, Tulkki V, Oresic M, Moraes CT, Pietiläinen K, et al. Mitochondrial myopathy induces a starvation-like response. Hum Mol Genet. 2010; 19:3948–58. 10.1093/hmg/ddq310 20656789

[48] Beenken A, Mohammadi M. The FGF family: biology, pathophysiology and therapy. Nat Rev Drug Discov. 2009; 8:235–53. 10.1038/nrd2792 19247306 PMC3684054

[49] Jenniskens GJ, Oosterhof A, Brandwijk R, Veerkamp JH, van Kuppevelt TH. Heparan sulfate heterogeneity in skeletal muscle basal lamina: demonstration by phage display-derived antibodies. J Neurosci. 2000; 20:4099–111. 10.1523/JNEUROSCI.20-11-04099.2000 10818145 PMC6772625

[50] Tan BK, Hallschmid M, Adya R, Kern W, Lehnert H, Randeva HS. Fibroblast growth factor 21 (FGF21) in human cerebrospinal fluid: relationship with plasma FGF21 and body adiposity. Diabetes. 2011; 60:2758–62. 10.2337/db11-0672 21926274 PMC3198100

[51] Hsuchou H, Pan W, Kastin AJ. The fasting polypeptide FGF21 can enter brain from blood. Peptides. 2007; 28:2382–6. 10.1016/j.peptides.2007.10.007 17996984 PMC2151924

[52] Henriques A, Kastner S, Chatzikonstantinou E, Pitzer C, Plaas C, Kirsch F, Wafzig O, Krüger C, Spoelgen R, Gonzalez De Aguilar JL, Gretz N, Schneider A. Gene expression changes in spinal motoneurons of the SOD1(G93A) transgenic model for ALS after treatment with G-CSF. Front Cell Neurosci. 2015; 8:464. 10.3389/fncel.2014.00464 25653590 PMC4299451

[53] Shadrach JL, Stansberry WM, Milen AM, Ives RE, Fogarty EA, Antonellis A, Pierchala BA. Translatomic analysis of regenerating and degenerating spinal motor neurons in injury and ALS. iScience. 2021; 24:102700. 10.1016/j.isci.2021.102700 34235408 PMC8246596

[54] Restelli LM, Oettinghaus B, Halliday M, Agca C, Licci M, Sironi L, Savoia C, Hench J, Tolnay M, Neutzner A, Schmidt A, Eckert A, Mallucci G, et al. Neuronal Mitochondrial Dysfunction Activates the Integrated Stress Response to Induce Fibroblast Growth Factor 21. Cell Rep. 2018; 24:1407–14. 10.1016/j.celrep.2018.07.023 30089252 PMC6092266

[55] Koene S, de Laat P, van Tienoven DH, Vriens D, Brandt AM, Sweep FC, Rodenburg RJ, Donders AR, Janssen MC, Smeitink JA. Serum FGF21 levels in adult m.3243A>G carriers: clinical implications. Neurology. 2014; 83:125–33. 10.1212/WNL.0000000000000578 24907231

[56] Dushay J, Chui PC, Gopalakrishnan GS, Varela-Rey M, Crawley M, Fisher FM, Badman MK, Martinez-Chantar ML, Maratos-Flier E. Increased fibroblast growth factor 21 in obesity and nonalcoholic fatty liver disease. Gastroenterology. 2010; 139:456–63. 10.1053/j.gastro.2010.04.054 20451522 PMC4862867

[57] Witzel S, Wagner M, Zhao C, Kandler K, Graf E, Berutti R, Oexle K, Brenner D, Winkelmann J, Ludolph AC. Fast versus slow disease progression in amyotrophic lateral sclerosis-clinical and genetic factors at the edges of the survival spectrum. Neurobiol Aging. 2022; 119:117–26. 10.1016/j.neurobiolaging.2022.07.005 35933239

[58] Dardiotis E, Siokas V, Sokratous M, Tsouris Z, Aloizou AM, Florou D, Dastamani M, Mentis AA, Brotis AG. Body mass index and survival from amyotrophic lateral sclerosis: A meta-analysis. Neurol Clin Pract. 2018; 8:437–44. 10.1212/CPJ.0000000000000521 30564498 PMC6276330

[59] Atassi N, Berry J, Shui A, Zach N, Sherman A, Sinani E, Walker J, Katsovskiy I, Schoenfeld D, Cudkowicz M, Leitner M. The PRO-ACT database: design, initial analyses, and predictive features. Neurology. 2014; 83:1719–25. 10.1212/WNL.0000000000000951 25298304 PMC4239834

[60] Traxinger K, Kelly C, Johnson BA, Lyles RH, Glass JD. Prognosis and epidemiology of amyotrophic lateral sclerosis: Analysis of a clinic population, 1997-2011. Neurol Clin Pract. 2013; 3:313–20. 10.1212/CPJ.0b013e3182a1b8ab 24195020 PMC3787117

[61] Lee I, Kazamel M, McPherson T, McAdam J, Bamman M, Amara A, Smith DL Jr, King PH. Fat mass loss correlates with faster disease progression in amyotrophic lateral sclerosis patients: Exploring the utility of dual-energy x-ray absorptiometry in a prospective study. PLoS One. 2021; 16:e0251087. 10.1371/journal.pone.0251087 33956876 PMC8101939

[62] Li JY, Sun XH, Cai ZY, Shen DC, Yang XZ, Liu MS, Cui LY. Correlation of weight and body composition with disease progression rate in patients with amyotrophic lateral sclerosis. Sci Rep. 2022; 12:13292. 10.1038/s41598-022-16229-9 35918363 PMC9345931

[63] Chiò A, Logroscino G, Hardiman O, Swingler R, Mitchell D, Beghi E, Traynor BG, and Eurals Consortium. Prognostic factors in ALS: A critical review. Amyotroph Lateral Scler. 2009; 10:310–23. 10.3109/17482960802566824 19922118 PMC3515205

[64] Moglia C, Calvo A, Grassano M, Canosa A, Manera U, D’Ovidio F, Bombaci A, Bersano E, Mazzini L, Mora G, Chiò A, and Piemonte and Valle d’Aosta Register for ALS (PARALS). Early weight loss in amyotrophic lateral sclerosis: outcome relevance and clinical correlates in a population-based cohort. J Neurol Neurosurg Psychiatry. 2019; 90:666–73. 10.1136/jnnp-2018-319611 30630957

[65] Jésus P, Fayemendy P, Nicol M, Lautrette G, Sourisseau H, Preux PM, Desport JC, Marin B, Couratier P. Hypermetabolism is a deleterious prognostic factor in patients with amyotrophic lateral sclerosis. Eur J Neurol. 2018; 25:97–104. 10.1111/ene.13468 28940704

[66] Steyn FJ, Ioannides ZA, van Eijk RP, Heggie S, Thorpe KA, Ceslis A, Heshmat S, Henders AK, Wray NR, van den Berg LH, Henderson RD, McCombe PA, Ngo ST. Hypermetabolism in ALS is associated with greater functional decline and shorter survival. J Neurol Neurosurg Psychiatry. 2018; 89:1016–23. 10.1136/jnnp-2017-317887 29706605 PMC6166607

[67] Dodge JC, Jensen EH, Yu J, Sardi SP, Bialas AR, Taksir TV, Bangari DS, Shihabuddin LS. Neutral Lipid Cacostasis Contributes to Disease Pathogenesis in Amyotrophic Lateral Sclerosis. J Neurosci. 2020; 40:9137–47. 10.1523/JNEUROSCI.1388-20.2020 33051352 PMC7673008

[68] Dodge JC, Treleaven CM, Fidler JA, Tamsett TJ, Bao C, Searles M, Taksir TV, Misra K, Sidman RL, Cheng SH, Shihabuddin LS. Metabolic signatures of amyotrophic lateral sclerosis reveal insights into disease pathogenesis. Proc Natl Acad Sci USA. 2013; 110:10812–7. 10.1073/pnas.1308421110 23754387 PMC3696768

[69] Dupuis L, Oudart H, René F, Gonzalez de Aguilar JL, Loeffler JP. Evidence for defective energy homeostasis in amyotrophic lateral sclerosis: benefit of a high-energy diet in a transgenic mouse model. Proc Natl Acad Sci USA. 2004; 101:11159–64. 10.1073/pnas.0402026101 15263088 PMC503756

[70] Steyn FJ, Li R, Kirk SE, Tefera TW, Xie TY, Tracey TJ, Kelk D, Wimberger E, Garton FC, Roberts L, Chapman SE, Coombes JS, Leevy WM, et al. Altered skeletal muscle glucose-fatty acid flux in amyotrophic lateral sclerosis. Brain Commun. 2020; 2:fcaa154. 10.1093/braincomms/fcaa154 33241210 PMC7677608

[71] Palamiuc L, Schlagowski A, Ngo ST, Vernay A, Dirrig-Grosch S, Henriques A, Boutillier AL, Zoll J, Echaniz-Laguna A, Loeffler JP, René F. A metabolic switch toward lipid use in glycolytic muscle is an early pathologic event in a mouse model of amyotrophic lateral sclerosis. EMBO Mol Med. 2015; 7:526–46. 10.15252/emmm.201404433 25820275 PMC4492815

[72] Maksimovic K, Youssef M, You J, Sung HK, Park J. Evidence of Metabolic Dysfunction in Amyotrophic Lateral Sclerosis (ALS) Patients and Animal Models. Biomolecules. 2023; 13:863. 10.3390/biom13050863 37238732 PMC10216611

[73] Solon-Biet SM, Cogger VC, Pulpitel T, Heblinski M, Wahl D, McMahon AC, Warren A, Durrant-Whyte J, Walters KA, Krycer JR, Ponton F, Gokarn R, Wali JA, et al. Defining the Nutritional and Metabolic Context of FGF21 Using the Geometric Framework. Cell Metab. 2016; 24:555–65. 10.1016/j.cmet.2016.09.001 27693377

[74] Lee I, Mitsumoto H, Lee S, Kasarskis E, Rosenbaum M, Factor-Litvak P, Nieves JW. Higher Glycemic Index and Glycemic Load Diet Is Associated with Slower Disease Progression in Amyotrophic Lateral Sclerosis. Ann Neurol. 2024; 95:217–29. 10.1002/ana.26825 37975189 PMC10842093

[75] Ma Y, Liu Z, Deng L, Du J, Fan Z, Ma T, Xiong J, Xiuyun X, Gu N, Di Z, Zhang Y. FGF21 attenuates neuroinflammation following subarachnoid hemorrhage through promoting mitophagy and inhibiting the cGAS-STING pathway. J Transl Med. 2024; 22:436. 10.1186/s12967-024-05239-y 38720350 PMC11077765

[76] Yang L, Nao J. Focus on Alzheimer’s Disease: The Role of Fibroblast Growth Factor 21 and Autophagy. Neuroscience. 2023; 511:13–28. 10.1016/j.neuroscience.2022.11.003 36372296

[77] Kakoty V, K C S, Tang RD, Yang CH, Dubey SK, Taliyan R. Fibroblast growth factor 21 and autophagy: A complex interplay in Parkinson disease. Biomed Pharmacother. 2020; 127:110145. 10.1016/j.biopha.2020.110145 32361164

[78] Sun Y, Wang Y, Chen ST, Chen YJ, Shen J, Yao WB, Gao XD, Chen S. Modulation of the Astrocyte-Neuron Lactate Shuttle System contributes to Neuroprotective action of Fibroblast Growth Factor 21. Theranostics. 2020; 10:8430–45. 10.7150/thno.44370 32724479 PMC7381735

[79] Van Damme P, Robberecht W. Clinical implications of recent breakthroughs in amyotrophic lateral sclerosis. Curr Opin Neurol. 2013; 26:466–72. 10.1097/WCO.0b013e328364c063 23945281

[80] Paez-Colasante X, Figueroa-Romero C, Sakowski SA, Goutman SA, Feldman EL. Amyotrophic lateral sclerosis: mechanisms and therapeutics in the epigenomic era. Nat Rev Neurol. 2015; 11:266–79. 10.1038/nrneurol.2015.57 25896087

[81] Shen Y, Zhu Z, Wang Y, Qian S, Xu C, Zhang B. Fibroblast growth factor-21 alleviates proteasome injury via activation of autophagy flux in Parkinson’s disease. Exp Brain Res. 2024; 242:25–32. 10.1007/s00221-023-06709-3 37910178 PMC10786996

[82] Teng YD, Mocchetti I, Wrathall JR. Basic and acidic fibroblast growth factors protect spinal motor neurones *in vivo* after experimental spinal cord injury. Eur J Neurosci. 1998; 10:798–802. 10.1046/j.1460-9568.1998.00100.x 9749747

[83] Teng YD, Mocchetti I, Taveira-DaSilva AM, Gillis RA, Wrathall JR. Basic fibroblast growth factor increases long-term survival of spinal motor neurons and improves respiratory function after experimental spinal cord injury. J Neurosci. 1999; 19:7037–47. 10.1523/JNEUROSCI.19-16-07037.1999 10436058 PMC6782862

[84] Delaye JB, Lanznaster D, Veyrat-Durebex C, Fontaine A, Bacle G, Lefevre A, Hergesheimer R, Lecron JC, Vourc’h P, Andres CR, Maillot F, Corcia P, Emond P, Blasco H. Behavioral, Hormonal, Inflammatory, and Metabolic Effects Associated with FGF21-Pathway Activation in an ALS Mouse Model. Neurotherapeutics. 2021; 18:297–308. 10.1007/s13311-020-00933-3 33021723 PMC8116478

[85] Ustyantseva E, Pavlova SV, Malakhova AA, Ustyantsev K, Zakian SM, Medvedev SP. Oxidative stress monitoring in iPSC-derived motor neurons using genetically encoded biosensors of H_2_O_2_. Sci Rep. 2022; 12:8928. 10.1038/s41598-022-12807-z 35624228 PMC9142597

[86] Mead RJ, Shan N, Reiser HJ, Marshall F, Shaw PJ. Amyotrophic lateral sclerosis: a neurodegenerative disorder poised for successful therapeutic translation. Nat Rev Drug Discov. 2023; 22:185–212. 10.1038/s41573-022-00612-2 36543887 PMC9768794

[87] Ji K, Zheng J, Lv J, Xu J, Ji X, Luo YB, Li W, Zhao Y, Yan C. Skeletal muscle increases FGF21 expression in mitochondrial disorders to compensate for energy metabolic insufficiency by activating the mTOR-YY1-PGC1α pathway. Free Radic Biol Med. 2015; 84:161–70. 10.1016/j.freeradbiomed.2015.03.020 25843656

[88] Liu X, Wang Y, Hou L, Xiong Y, Zhao S. Fibroblast Growth Factor 21 (FGF21) Promotes Formation of Aerobic Myofibers via the FGF21-SIRT1-AMPK-PGC1α Pathway. J Cell Physiol. 2017; 232:1893–906. 10.1002/jcp.25735 27966786

[89] Ribas F, Villarroya J, Hondares E, Giralt M, Villarroya F. FGF21 expression and release in muscle cells: involvement of MyoD and regulation by mitochondria-driven signalling. Biochem J. 2014; 463:191–9. 10.1042/BJ20140403 25055037

[90] Scaramozza A, Marchese V, Papa V, Salaroli R, Sorarù G, Angelini C, Cenacchi G. Skeletal muscle satellite cells in amyotrophic lateral sclerosis. Ultrastruct Pathol. 2014; 38:295–302. 10.3109/01913123.2014.937842 25079897

[91] Manzano R, Toivonen JM, Calvo AC, Oliván S, Zaragoza P, Rodellar C, Montarras D, Osta R. Altered *in vitro* proliferation of mouse SOD1-G93A skeletal muscle satellite cells. Neurodegener Dis. 2013; 11:153–64. 10.1159/000338061 22797053

[92] Martini M, Dobrowolny G, Aucello M, Musarò A. Postmitotic Expression of SOD1(G93A) Gene Affects the Identity of Myogenic Cells and Inhibits Myoblasts Differentiation. Mediators Inflamm. 2015; 2015:537853. 10.1155/2015/537853 26491230 PMC4600922

[93] Allodi I, Montañana-Rosell R, Selvan R, Löw P, Kiehn O. Locomotor deficits in a mouse model of ALS are paralleled by loss of V1-interneuron connections onto fast motor neurons. Nat Commun. 2021; 12:3251. 10.1038/s41467-021-23224-7 34059686 PMC8166981

[94] Frey D, Schneider C, Xu L, Borg J, Spooren W, Caroni P. Early and selective loss of neuromuscular synapse subtypes with low sprouting competence in motoneuron diseases. J Neurosci. 2000; 20:2534–42. 10.1523/JNEUROSCI.20-07-02534.2000 10729333 PMC6772256

[95] Yan J, Nie Y, Cao J, Luo M, Yan M, Chen Z, He B. The Roles and Pharmacological Effects of FGF21 in Preventing Aging-Associated Metabolic Diseases. Front Cardiovasc Med. 2021; 8:655575. 10.3389/fcvm.2021.655575 33869312 PMC8044345

[96] Cardoso AL, Fernandes A, Aguilar-Pimentel JA, de Angelis MH, Guedes JR, Brito MA, Ortolano S, Pani G, Athanasopoulou S, Gonos ES, Schosserer M, Grillari J, Peterson P, et al. Towards frailty biomarkers: Candidates from genes and pathways regulated in aging and age-related diseases. Ageing Res Rev. 2018; 47:214–77. 10.1016/j.arr.2018.07.004 30071357

[97] Pandya VA, Patani R. Decoding the relationship between ageing and amyotrophic lateral sclerosis: a cellular perspective. Brain. 2020; 143:1057–72. 10.1093/brain/awz360 31851317 PMC7174045

[98] Cortes-Araya Y, Stenhouse C, Salavati M, Dan-Jumbo SO, Ho W, Ashworth CJ, Clark E, Esteves CL, Donadeu FX. KLB dysregulation mediates disrupted muscle development in intrauterine growth restriction. J Physiol. 2022; 600:1771–90. 10.1113/JP281647 35081669 PMC9303651

[99] Larson KR, Jayakrishnan D, Soto Sauza KA, Goodson ML, Chaffin AT, Davidyan A, Pathak S, Fang Y, Gonzalez Magaña D, Miller BF, Ryan KK. FGF21 Induces Skeletal Muscle Atrophy and Increases Amino Acids in Female Mice: A Potential Role for Glucocorticoids. Endocrinology. 2024; 165:bqae004. 10.1210/endocr/bqae004 38244215 PMC10849119

[100] Oost LJ, Kustermann M, Armani A, Blaauw B, Romanello V. Fibroblast growth factor 21 controls mitophagy and muscle mass. J Cachexia Sarcopenia Muscle. 2019; 10:630–42. 10.1002/jcsm.12409 30895728 PMC6596457

[101] Tezze C, Romanello V, Desbats MA, Fadini GP, Albiero M, Favaro G, Ciciliot S, Soriano ME, Morbidoni V, Cerqua C, Loefler S, Kern H, Franceschi C, et al. Age-Associated Loss of OPA1 in Muscle Impacts Muscle Mass, Metabolic Homeostasis, Systemic Inflammation, and Epithelial Senescence. Cell Metab. 2017; 25:1374–89.e6. 10.1016/j.cmet.2017.04.021 28552492 PMC5462533

[102] Dunshee DR, Bainbridge TW, Kljavin NM, Zavala-Solorio J, Schroeder AC, Chan R, Corpuz R, Wong M, Zhou W, Deshmukh G, Ly J, Sutherlin DP, Ernst JA, Sonoda J. Fibroblast Activation Protein Cleaves and Inactivates Fibroblast Growth Factor 21. J Biol Chem. 2016; 291:5986–96. 10.1074/jbc.M115.710582 26797127 PMC4786731

