# Supplementary material for: The myokine FGF21 associates with enhanced survival in ALS and mitigates stress-induced cytotoxicity
Source: Aging (Albany NY). 2025 Aug 9;17(8):2033–62. doi: 10.18632/aging.206298 (PMC12422826; doi:10.18632/aging.206298)
Supplement: Supplementary Figures [file aging-17-8-206298-s001.pdf]

SUPPLEMENTARY FIGURES

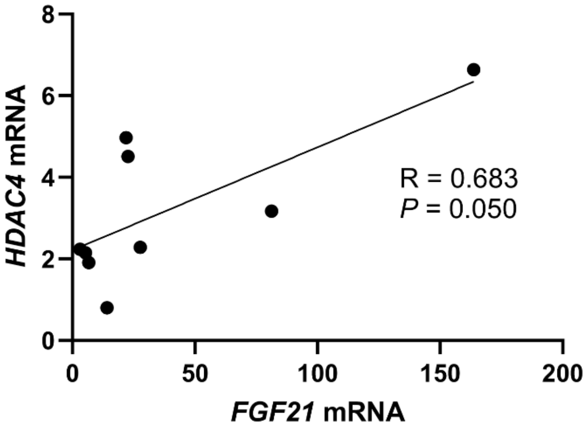

Supplementary Figure 1. Spearman rank test correlation between *HDAC4* and *FGF21* mRNA expression levels (assessed by qPCR) in 9 post-mortem ALS muscle samples.

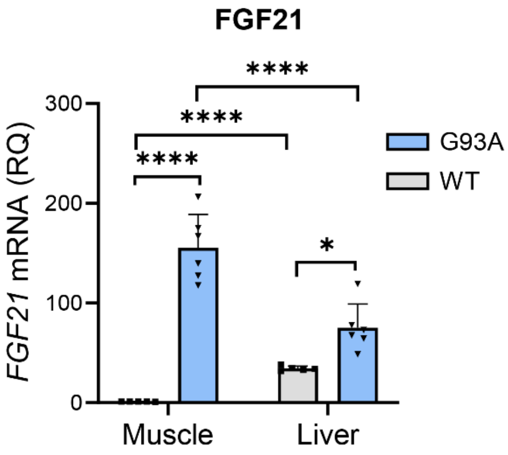

Supplementary Figure 2. FGF21 mRNA is increased in muscle and liver tissue in the SOD1^{G93A} mouse. RNA was extracted from wild-type (WT) and SOD1^{G93A} mouse tissue at post-natal day 60 and assessed by qPCR for FGF21 mRNA. All values represent fold-change compared to WT muscle which was set at 1. Data points represent individual mice and bars represent the mean ± SD. **P* < 0.05, *****P* < 0.0001; one-way ANOVA followed by Tukey's multiple comparisons test.

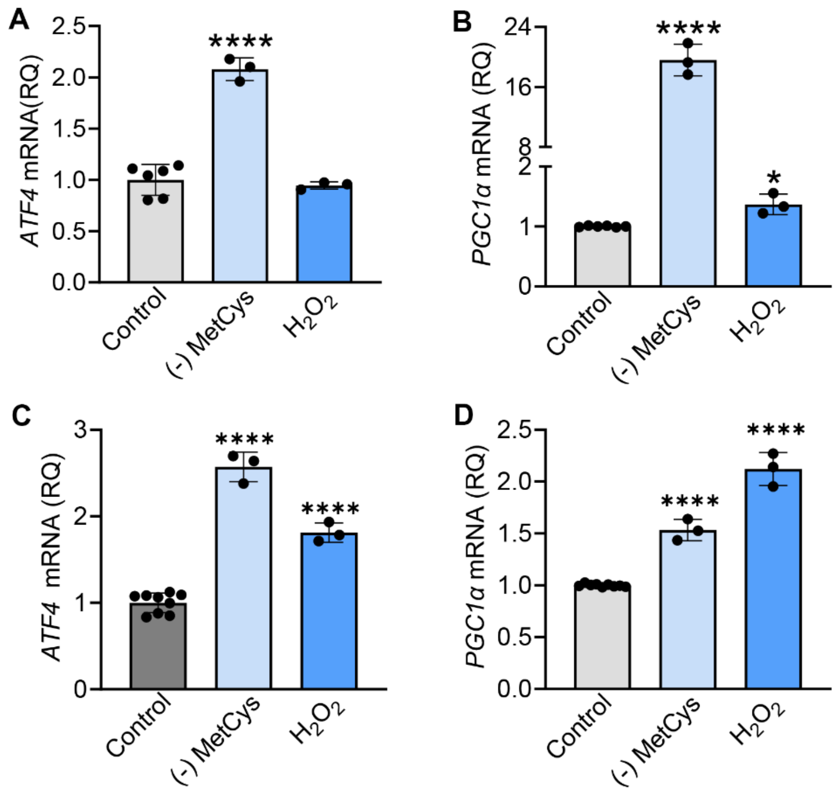

Supplementary Figure 3. Oxidative stressors induce *ATF4* and *PGC-1α* in NSC-34 cells and C2C12 myoblasts. (**A**, **B**) *ATF4* and *PGC-1α* mRNA levels in NSC-34 cells were quantified after treatment with 100 µM H_2O_2 or methionine-cysteine (MetCys)-depleted media for 24 h. (**C**, **D**) *ATF4* and *PGC-1α* mRNA levels were quantified in C2C12 myoblasts after exposure to the same conditions as in (**A**, **B**). Data points are independent biological samples and bars represent the mean ± SD.*P = 0.048, ****P < 0.0001; one-way ANOVA followed by Tukey's multiple comparisons test.

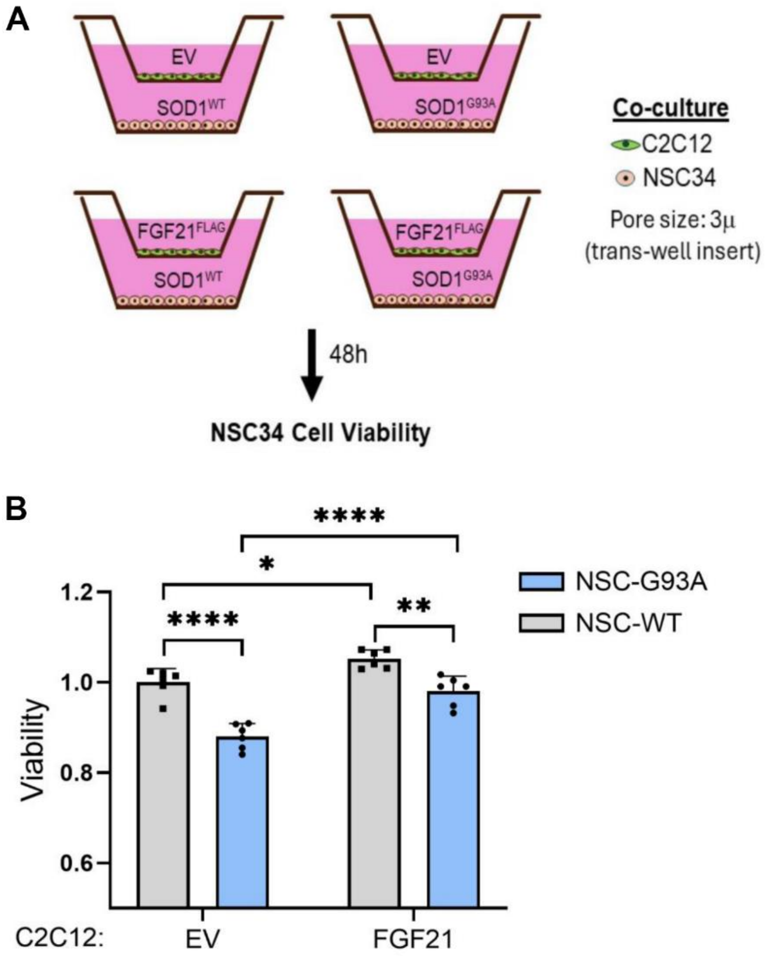

Supplementary Figure 4. C2C12 cells expressing FGF21 rescue SOD1^{G93A}-mediated toxicity in NSC-34 motor neuron like cells in co-culture. (**A**) Schematic of co-culture system used. (**B**) Cell viability of NSC-34 cells expressing either WT-SOD1 or SOD1^{G93A} (lower well) was assessed in the presence or absence of FGF21-expressing C2C12 cells (upper well). Data points represent biological replicates and bars are the mean ± SD. *P = 0.025, **P = 0.002, ****P < 0.0001; one-way ANOVA followed by Tukey's multiple comparisons test.

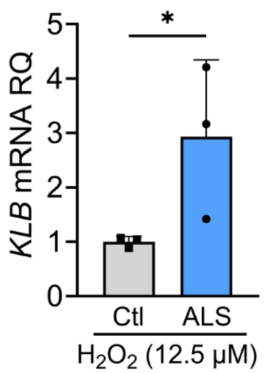

Supplementary Figure 5. H_2O_2 treatment induces *KLB* mRNA in motor neurons derived from C9-ALS patients. iPSC-derived motor neurons from normal and ALS patient donors were exposed to 12.5 μM of H_2O_2 for 24 h, and *KLB* mRNA levels were quantified using qRT-PCR. Bars represent the mean ± SD of 3 independent biological samples. *P = 0.03; unpaired one-tailed t-test.

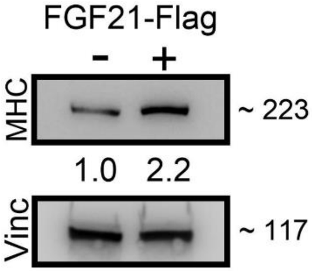

Supplementary Figure 6. FGF21 increases the levels of MHC protein during myogenesis. C2C12 myoblasts were transfected with an FGF21-FLAG plasmid and cultured in DM for 96 h. Cells were lysed and the protein lysates were immunoblotted with antibodies against MHC and vinculin. For densitometry values, empty vector (- FGF21-FLAG) was set at 1.

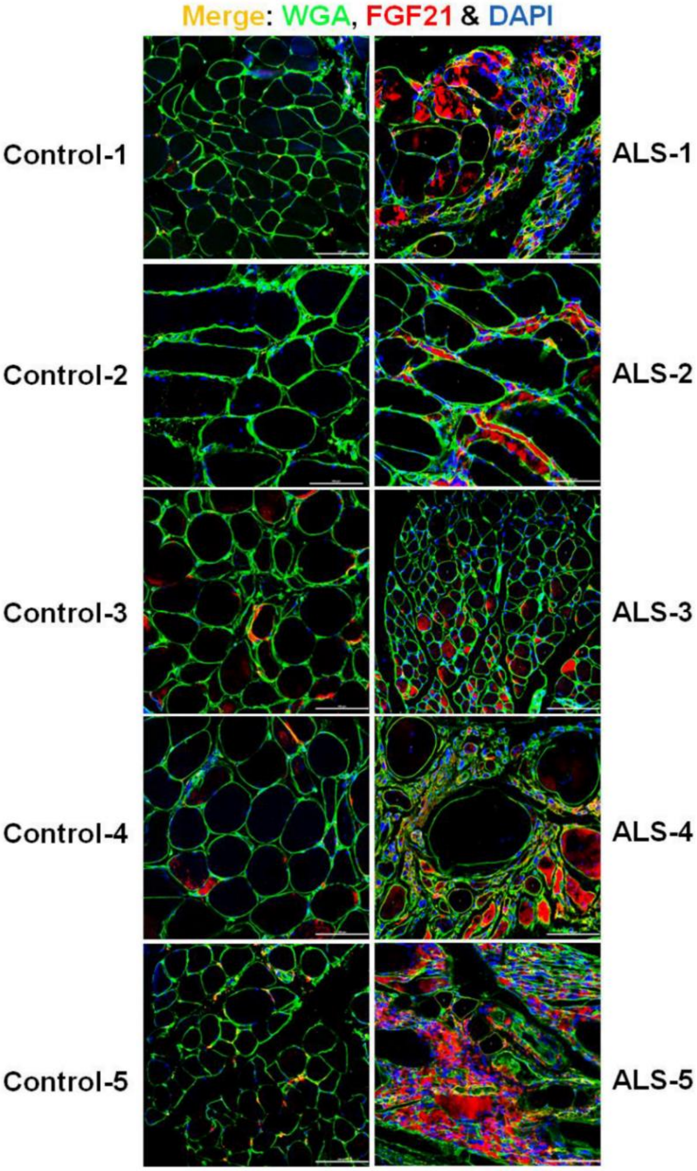

Supplementary Figure 7. FGF21 localizes to atrophic myofibers in ALS muscle. Tissue sections from five ALS patients and five normal controls were immunostained with an anti-FGF21 antibody (red) and counterstained with DAPI (blue) and WGA (green). Intense immunoreactivity is observed in atrophic myofibers and in the endomysial space in the ALS muscle sections. Scale bars, 100 µM.
