# Supplementary material for: The myokine FGF21 associates with enhanced survival in ALS and mitigates stress-induced cytotoxicity
Source: Aging (Albany NY). 2025 Aug 9;17(8):2033–62. doi: 10.18632/aging.206298 (PMC12422826; doi:10.18632/aging.206298)
Supplement: Supplementary Tables [file aging-17-8-206298-s002.pdf]

## SUPPLEMENTARY TABLES

**Supplementary Table 1. Demographic and clinical data for iPSC-derived motor neurons.**

| Cell Line | Sex | Age (y) | Clinical diagnosis | Primary tissue | Mutation |
|---|---|---|---|---|---|
| **CS0002iCTR** | M | 51 | Normal | PBMC | N/A |
| **CS83iCTR** | F | 21 | Normal | Fibroblast | N/A |
| **CS188iCTR** | M | 80 | Normal | PBMC | N/A |
| **CS14iCTR** | F | 35 | Normal | Fibroblast | N/A |
| **CS00iCTR** | M | 6 | Normal | Fibroblast | N/A |
| **FA0000011** | F | 49 | Normal | Fibroblast | N/A |
| **NN0003920** | M | 64 | Normal | Fibroblast | N/A |
| **CS0118iALS-SOD1-I114T** | F | 73 | ALS | Fibroblast | SOD1 I113T |
| **CS28iALS** | M | 47 | ALS | Fibroblast | C9ORF72 (HRE ~800) |
| **CS29iALS** | M | 47 | ALS | Fibroblast | C9ORF72 (HRE ~800) |
| **CS52iALS** | M | 49 | ALS | Fibroblast | C9ORF72 (HRE ~800) |
| **CS30iALS** | F | 51 | ALS | Fibroblast | C9ORF72 (HRE ~70) |
| **NN0004306** | F | 51 | ALS | Fibroblast F10330 | C9ORF72 (HRE 2.7kb) |
| **NN0004307** | M | 57 | ALS | Fibroblast F09152 | C9ORF72 (HRE 6-8kb) |

ALS, amyotrophic lateral sclerosis; F, female; HRE, hexanucleotide repeat expansion; M, male; ORF, open reading frame; PBMC, peripheral blood mononuclear cells; SOD1, superoxide dismutase 1; y, years.

**Supplementary Table 2. Demographic and clinical data of tissue samples.**

| | Biopsy | | Autopsy | |
|---|---|---|---|---|
| | **Normal** | **ALS** | **Normal** | **ALS** |
| **Number** | 24 | 36 | 22 | 23 |
| **Mean age (years)** [a] | 52 ± 15 | 57 ± 13 | 67 ± 12 | 64 ± 11 |
| **Age range (years)** | 24 - 77 | 27- 86 | 34 - 83 | 40 - 81 |
| **Gender (M:F)** | 11:13 | 21:15 | 18:4 | 18:5 |
| **Duration** [b] **(m)** | | 15 ± 9 | | 51 ± 32 |
| **Diagnosis** | | Spinal onset (33 ) | | Spinal onset (20 ) |
| | | Bulbar onset (3) | | Bulbar onset (3) |
| **Muscle sampled** | | | | |
| **Biceps brachii** | 5 | 2 | | 2 |
| **Deltoid** | 3 | 11 | 4 | 3 |
| **Vastus lateralis** | 15 | 8 | 3 | 5 |
| **Tibialis anterior** | 1 | 15 | | Triceps (1) |

[a]Mean age (± SD) at time of sample collection.
[b]Mean duration (± SD) from onset of symptoms to sample collection. Duration was unknown for three ALS patients in the biopsy pool.

**Supplementary Table 3. Demographic and clinical data of plasma samples.**

| | Normal | ALS |
|---|---|---|
| Number | 23 | 28 |
| Mean age (y) [a] | 61 ± 9 | 59 ± 10 |
| Age range (y) | 45 - 84 | 35- 82 |
| Gender (M:F) | 12:11 | 18:10 |
| Duration [b] (m) | | 26 ± 20 |
| Onset | | Spinal onset (22)<br>Bulbar onset (6) |

F, female; M, male; m, months; y, years.
[a]Mean age (± SD) at time of sample collection.
[b]Mean duration (± SD) from onset of symptoms to sample collection.

**Supplementary Table 4. ALS study patients.**

| Plasma FGF21 (FC)[a] | < 1.5 | ≥ 1.5 |
|---|---|---|
| Number | 7 | 9 |
| Age (y)[b] | 65 ± 9 | 57 ± 9[d] |
| Age range (y) | 58 - 82 | 42 – 70[d] |
| Gender (M:F) | 4:3 | 7:2 |
| Duration[c] (m) | 18 ± 10 | 26 ± 23[d] |
| Onset | Bulbar (4)<br>Spinal (3) | Spinal (9) |

F, female; FC, fold-change; M, male; m, months; y, years.
[a]Fold-change over normal control group.
[b]Mean age (± SD) at time of sample collection.
[c] Mean duration (± SD) from onset of symptoms to sample collection.
[d]No significant difference between the 2 groups.
